# Hyperlipidemia Aggravates Alveolar Bone Loss via Periodontal Ligament Stem Cell Ferroptosis Through GSK3β Dependent Ubiquitin‐Mediated NRF2 Degradation

**DOI:** 10.1002/advs.75157

**Published:** 2026-04-07

**Authors:** Yuxiao Zhang, Xiangyao Wang, Yaxin Wu, Liping Liu, Gaoshaer Nuerlan, Ahsawle Ozathaley, Xiaorui Zhang, Jinping Wang, Bowen Yang, Jing Mao, Yan Liu, Shiqiang Gong

**Affiliations:** ^1^ Department of Stomatology Tongji Hospital Tongji Medical College Huazhong University of Science and Technology Wuhan China; ^2^ School of Stomatology Tongji Medical College Huazhong University of Science and Technology & Hubei Province Key Laboratory of Oral and Maxillofacial Development and Regeneration Wuhan China; ^3^ Central Laboratory Department of Orthodontics Peking University School and Hospital for Stomatology Beijing China; ^4^ Beijing Advanced Center of Cellular Homeostasis and Aging‐Related Diseases Institute of Advanced Clinical Medicine Peking University Beijing China

**Keywords:** bone homeostasis, ferroptosis, hyperlipidemia, periodontal ligament stem cells

## Abstract

Oxidative stress is increasingly recognized as a key contributor to the pathophysiology of periodontitis, particularly in patients with metabolic disturbances such as hyperlipidemia. The osteogenesis of periodontal ligament stem cells (PDLSCs) plays a pivotal role in maintaining alveolar bone homeostasis. In this study, we found that dysregulated lipid metabolism induces ferroptosis and mitochondrial dysfunction in PDLSCs, impairing their osteogenic differentiation and exacerbating alveolar bone loss in both in vitro and in vivo models. Mechanistically, free fatty acids activate glycogen synthase kinase 3 beta (GSK3β), which facilitates Kelch‐like ECH‐associated protein 1 (KEAP1)‐independent, beta‐transducin repeat‐containing protein (β‐TrCP)‐mediated ubiquitin–proteasome degradation of nuclear factor erythroid 2‐related factor 2 (NRF2). This leads to reduced expression of key antioxidant enzymes such as Glutathione peroxidase 4 (GPX4) and solute carrier family 7 member 11 (SLC7A11), resulting in redox imbalance and ferroptosis of PDLSCs. Notably, pharmacological inhibition of either GSK3β or ferroptosis restores NRF2 stability, alleviates oxidative stress, and rescues the osteogenic potential of PDLSCs. Furthermore, local inhibition of GSK3β significantly attenuates alveolar bone destruction in a hyperlipidemia‐associated periodontitis mouse model. Collectively, our findings identify a novel GSK3β–NRF2–ferroptosis pathway that mediates the detrimental effects of hyperlipidemia on PDLSC function and periodontal homeostasis, offering a promising therapeutic target for metabolic disorder‐associated periodontal damage.

## Graphical abstract

Lipid metabolic stress triggers ferroptosis in PDLSCs through the GSK3β/NRF2 pathway, thereby aggravating periodontal bone loss. Upregulated GSK3β promotes NRF2 ubiquitination and proteasomal degradation via β‐TrCP, suppressing NRF2 nuclear translocation and antioxidant target expression. Consequently, ROS, Fe^2^^+^, and lipid peroxidation accumulate, impairing osteogenic differentiation and driving ferroptotic cell death.

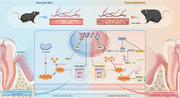

## 1 Introduction

Periodontitis is a prevalent chronic inflammatory disease characterized by the progressive destruction of the periodontal ligament and alveolar bone [1, 2]. Its pathogenesis is multifactorial, involving microbial dysbiosis, systemic inflammation, and lifestyle‐related risk factors, and can be further aggravated by metabolic alterations [3, 4]. Emerging evidence suggests an intimate association between periodontitis and components of metabolic syndrome (MetS), particularly obesity and hyperlipidemia [5]. Clinical evidence has validated that individuals with MetS exhibit a higher prevalence and greater severity of periodontal lesions compared to metabolically healthy populations [6, 7]. Notably, MetS has been formally recognized as a modifying factor that exacerbates periodontal tissue destruction and increases the risk of periodontitis, as reflected in the 2017 World Workshop classification of periodontal diseases [8, 9]. One of the key shared pathological mechanisms underpinning this association is oxidative stress [10, 11]. Under metabolic dysregulation, excessive lipid accumulation in the tissue microenvironment may disrupt intracellular redox homeostasis, elevate reactive oxygen species (ROS) levels, and ultimately compromise periodontal tissue integrity [12]. However, the underlying mechanism on how MetS aggravates periodontal bone loss requires in‐depth dissection.

Ferroptosis, a recently identified form of regulated cell death distinct from apoptosis or necrosis, is characterized by the accumulation of toxic lipid peroxides on cell membranes and has been implicated in various diseases [13]. Notably, ferroptosis is closely linked to metabolic disorders involving lipid dysregulation. For instance, obesity and diabetes are associated with systemic iron overload and lipid peroxidation, creating a microenvironment conducive to ferroptosis in affected tissues [14, 15]. The biochemical hallmarks of ferroptosis include excessive lipid peroxidation and elevated intracellular ROS [16]. Glutathione peroxidase 4 (GPX4), a selenoenzyme, plays a crucial protective role by reducing phospholipid hydroperoxides into non‐toxic lipid alcohols, thereby preventing cells from ferroptosis [17]. Another key regulator of ferroptosis sensitivity is nuclear factor erythroid 2‐related factor 2 (NRF2), a transcription factor that orchestrates cellular antioxidant defense system. NRF2 drives the expression of numerous genes involved in glutathione synthesis, iron sequestration, and lipid antioxidant production, including GPX4 [18]. Under homeostatic conditions, NRF2 helps to maintain redox balance and prevents excessive lipid peroxidation. Conversely, dysfunction of NRF2 leads to uncontrolled oxidative damage and ferroptosis [19].

Periodontal ligament stem cells (PDLSCs), residing within the periodontal ligament, are a subset of mesenchymal stem cells (MSCs) that play an essential role in maintaining periodontal homeostasis and bone remodeling [20]. However, their biological function is highly susceptible to pathological stimuli such as chronic inflammation, oxidative stress, lipid peroxidation, and mechanical overload. Aberrant external cues disrupt osteogenesis‐related gene expression in PDLSCs while favoring adipogenic or osteoclastogenic pathways, ultimately impairing their bone‐forming capacity and leading to periodontal bone loss in the scenario of periodontitis [21, 22, 23]. Thus, the impaired osteogenesis of PDLSCs is a fundamental driver of alveolar bone destruction in chronic periodontitis and a better understanding of the underlying mechanism is crucial for identifying novel targets for periodontal bone regeneration.

**SCHEME 1:**
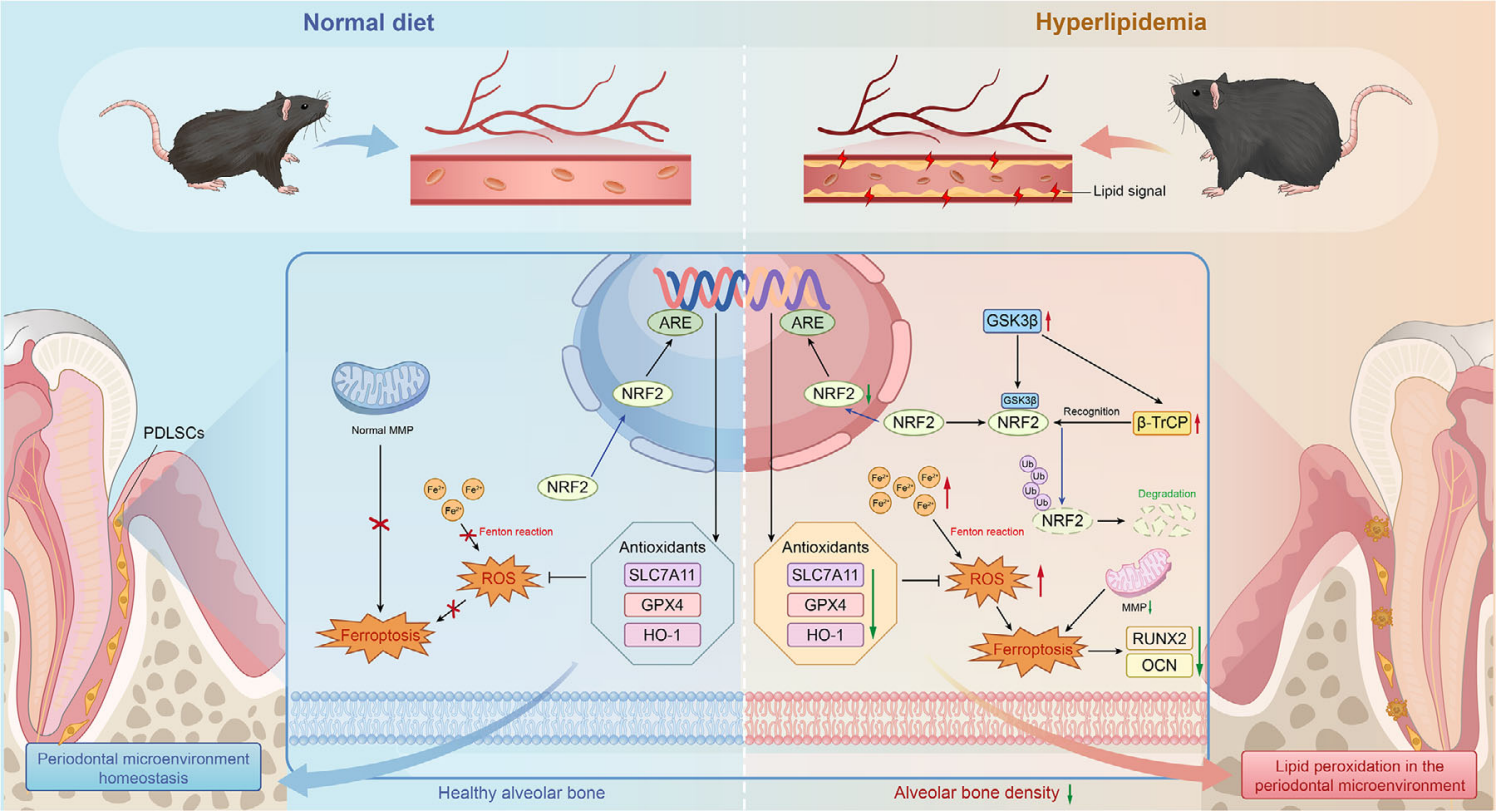
Schematic illustration of the mechanism by which lipid metabolic stress induces ferroptosis in PDLSCs via the GSK3β/NRF2 pathway, aggravating periodontal bone loss. Under lipid metabolic stress, the expression of GSK3β is upregulated in PDLSCs, enhancing its interaction with NRF2. This interaction facilitates the recognition and ubiquitination of NRF2 by β‐TrCP, leading to its degradation via the proteasome pathway. As a result, nuclear translocation of NRF2 is suppressed, downregulating the transcription of its downstream antioxidant and anti‐ferroptotic target molecules, such as GPX4, HO‐1, and SLC7A11. This leads to increased lipid peroxidation, intracellular accumulation of ROS and Fe^2^^+^, ultimately inducing ferroptosis and impairing the osteogenic differentiation capacity of PDLSCs.

In this study, we revealed the mechanistic link between hyperlipidemia and ferroptosis of PDLSCs within the context of periodontitis. Under hyperlipidemic condition, reduced periodontal bone mass was accompanied by ferroptosis of PDLSCs. Our study confirmed that inhibition of ferroptosis mitigates inflammatory alveolar bone loss under hyperlipidemic condition. As illustrated in Scheme 1, hyperlipidemia activates glycogen synthase kinase 3 beta (GSK3β), which interacts with NRF2 and promotes NRF2 ubiquitination and proteasomal degradation. The resulting reduction of NRF2 stability weakens antioxidant defenses in PDLSCs, leading to diminished expression of protective enzymes such as GPX4. This impairment exacerbates lipid peroxidation and ROS accumulation, ultimately triggering ferroptotic cell death. These findings unveil a previously unrecognized mechanism by which lipid metabolic dysregulation promotes periodontal damage through induction of PDLSC ferroptosis. More importantly, our in vivo rescue experiment validated the GSK3β–NRF2 axis as a druggable target for regeneration therapy of periodontitis under hyperlipidemic condition.

## 2 Results

### 2.1 High Fat Diet Impairs PDLSC Osteogenic Differentiation and Compromises Alveolar Bone Quality in Mice

**FIGURE 1:**
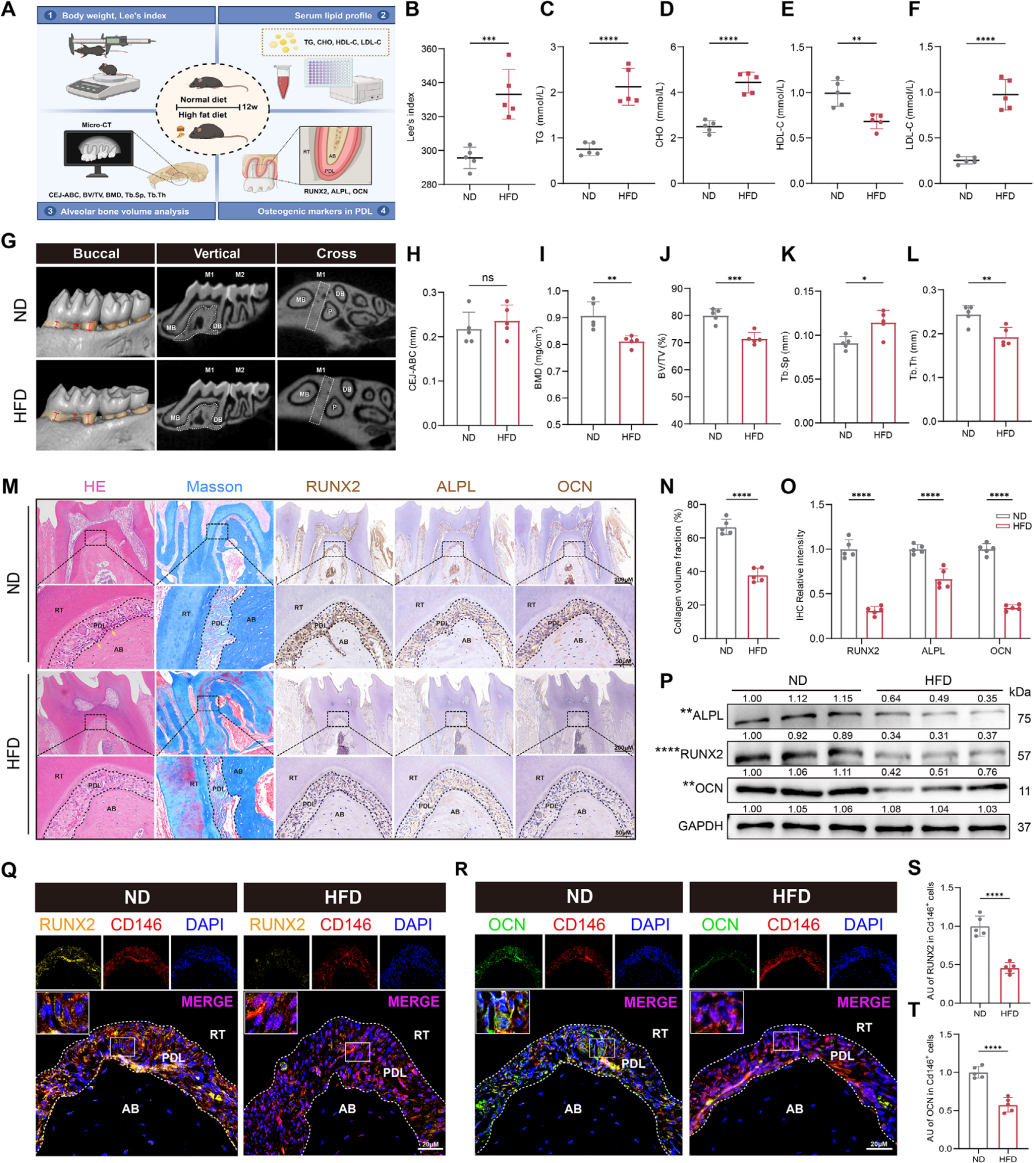
High fat diet impaired alveolar bone quality and osteogenic differentiation of periodontal ligament stem cells (PDLSCs) in mice. (A) Schematic illustration of the experimental design. (n = 5/group). (B) Lee's index after 12 weeks of diet intervention. (C–F) Serum biochemical analysis of TG, CHO, LDL‐C, and HDL‐C after 12 weeks of feeding. (G) Representative micro‐CT images of alveolar bone in buccal, vertical, and cross‐sectional views. (H–L) Quantitative analysis of CEJ–ABC distance, bone mineral density (BMD), bone volume/total volume (BV/TV), trabecular separation (Tb.Sp), and trabecular thickness (Tb.Th). (M) Representative images of HE, Masson's trichrome, and immunohistochemical staining for RUNX2, ALPL, and OCN in the PDL. (N) Quantification of collagen content in the PDL based on Masson staining. (O) Quantification of IHC staining intensity for RUNX2, ALPL, and OCN. (P) Western blot for protein expression of ALPL, RUNX2, and OCN in PDL tissues and corresponding quantitative analysis of the heat map. (Q,R) Representative immunofluorescence images showing co‐staining of CD146 with RUNX2 or OCN. (S,T) Quantification of the mean fluorescence intensity of RUNX2 or OCN in CD146^+^ cells. (A–O, Q–T: n = 5 per group; P: n = 3 per group; values represented mean ± SD; ns indicates no significant, ^*^ *p* < 0.05, ^**^ *p* < 0.01, ^***^ *p* < 0.001, ^****^ *p* < 0.0001).

To establish a metabolic model mimicking human hyperlipidemia, we first subjected C57BL/6J mice to a high fat diet (HFD) (Figure 1A). After 12 weeks of HFD feeding, mice displayed significantly increased body weight and Lee's index compared to those fed a normal diet (ND) (Figure 1B; Figure S1). Serum biochemical analysis revealed significantly elevated levels of triglycerides (TG), total cholesterol (CHO), and low‐density lipoprotein cholesterol (LDL‐C), accompanied by a marked reduction in high‐density lipoprotein cholesterol (HDL‐C) (Figure 1C–F). These changes are characteristic of mixed hyperlipidemia and reflect substantial disturbances in lipid metabolism, closely resembling the metabolic alterations observed in humans with long‐term consumption of high fat, high oil diets.

Micro‐CT analysis further revealed that, although the distance from the cementoenamel junction to the alveolar bone crest (CEJ–ABC) remained largely unchanged, HFD mice exhibited significantly reduced bone mineral density (BMD), bone volume fraction (BV/TV), and trabecular thickness (Tb. Th), along with a marked increase in trabecular separation (Tb. Sp), indicative of deteriorated alveolar bone microarchitecture (Figure 1G–L). Given the critical role of the periodontal ligament (PDL) in regulating alveolar bone remodeling, we next assessed histological changes in this region. In HFD mice, the PDL exhibited disorganized cell alignment and decreased cellularity along the alveolar bone surface (highlighted by yellow arrows), while Masson's trichrome staining showed a notable reduction in collagen deposition within the PDL (Figure 1M,N).

Immunohistochemistry and Western blot analysis further demonstrated that the expression levels of osteogenic markers—RUNX2, ALPL, and OCN—were significantly downregulated in the PDL of HFD mice (Figure 1M,O,P). Moreover, immunofluorescence co‐staining of CD146, a mesenchymal stem cell marker widely used to identify PDLSCs in the periodontal ligament region, with osteogenic markers revealed diminished expression of RUNX2 and OCN specifically within the periodontium in the HFD group (Figure 1Q–T) [2, 11]. Together, these results suggest that chronic HFD exposure compromises the osteogenic differentiation capacity of PDLSCs, contributing to the observed reduction in alveolar bone mass and quality in HFD mice.

### 2.2 High Fat Diet Induces Lipid Peroxidation and Ferroptosis‐Related Changes in the Periodontal Ligament

**FIGURE 2:**
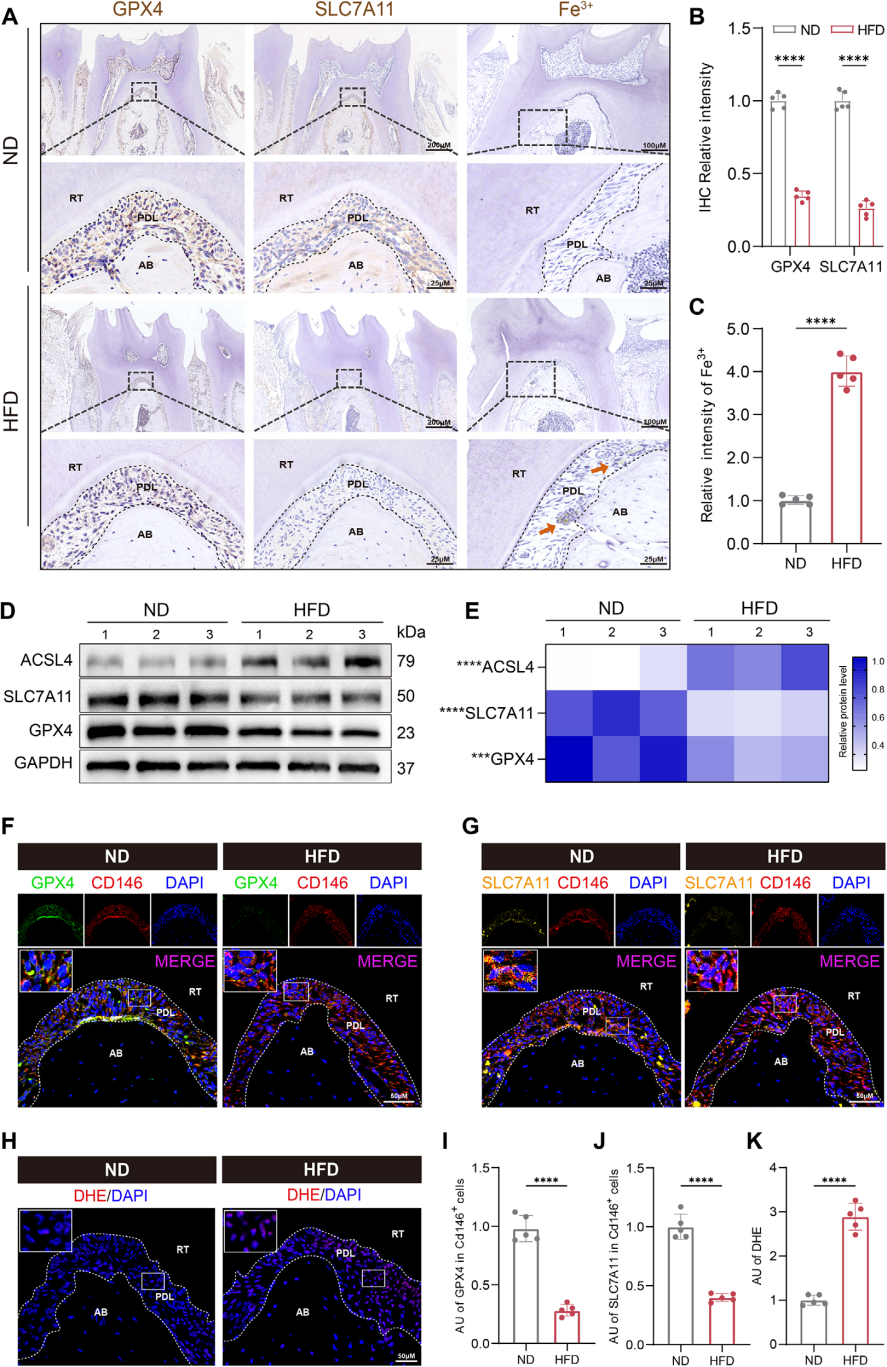
High fat diet induces lipid peroxidation in the periodontal ligament and activates ferroptosis in PDLSCs. (A) Immunohistochemical staining of GPX4, SLC7A11, and Fe^3^^+^ in the PDL of mice under normal diet and HFD. (B) Quantification of IHC staining intensity for GPX4 and SLC7A11. (C) Quantification of Fe^3^^+^ deposition intensity. (D,E) Western blot analysis and corresponding heatmap quantification of ACSL4, SLC7A11, and GPX4 protein expression in PDL tissues. (F,G) Immunofluorescence co‐staining of the mesenchymal stem cell marker CD146 with SLC7A11 or GPX4 in the PDL region. (H) Representative images of DHE staining indicating ROS levels in the PDL. (I,J) Quantification of the mean fluorescence intensity of GPX4 or SLC7A11 in CD146^+^ cells. (K) Quantification of fluorescence intensity for DHE. (A–C, F–K: n = 5 per group; D–E: n = 3 per group; values represented mean ± SD; ns indicates no significant, ^*^ *p* < 0.05, ^**^ *p* < 0.01, ^***^ *p* < 0.001, ^****^ *p* < 0.0001).

In addition to the downregulation of osteogenic markers in the PDL region of HFD mice, we observed a significant reduction in the expression of key ferroptosis‐regulating molecules GPX4 and SLC7A11 (Figure 2A,B). These proteins are critical for maintaining cellular redox homeostasis and protecting against lipid peroxidation‐induced cell death [24]. Their downregulation was accompanied by increased deposition of ferric ions (Fe^3^^+^), a hallmark of ferroptotic activity (Figure 2A,C). Western blot analysis of isolated PDL tissue further validated these molecular alterations (Figure 2D,E). Consistently, HFD mice exhibited markedly elevated levels of reactive oxygen species (ROS) within the PDL, indicating oxidative stress (Figure 2H,K). To further investigate whether ferroptosis was activated in PDLSCs, we performed immunofluorescence staining using the mesenchymal stem cell marker CD146, along with the lipid peroxidation‐related proteins SLC7A11 and GPX4. The colocalization and reduced expression of these markers in PDLSCs under HFD conditions strongly support the activation of the ferroptosis pathway in response to high fat‐induced metabolic stress (Figure 2F,G,I,J).

### 2.3 Free Fatty Acids Induce Ferroptosis of PDLSCs In Vitro

The above findings suggest that dysregulated lipid metabolism induces ferroptosis in PDLSCs, thereby impairing their osteogenic differentiation potential. To further investigate this mechanism, human PDLSCs (hPDLSCs) were isolated from extracted teeth of healthy volunteers. These cells exhibited typical MSC‐like characteristics, including multilineage differentiation potential and the expression of specific surface markers. hPDLSCs were obtained by scraping periodontal ligament tissues and expanding them in culture, forming dense fibroblast‐like colonies (Figure S2A). Under appropriate induction conditions, osteogenic, adipogenic, and chondrogenic differentiation were successfully achieved and verified by Alizarin Red S, Oil Red O, and Alcian Blue staining, respectively, revealing the formation of calcium nodules, lipid droplets, and cartilage‐like spheroid structures, thereby confirming their tri‐lineage differentiation capacity (Figure S2B). Flow cytometry further confirmed their MSC phenotype, showing positive expression of CD73, CD90, and CD105, and negative expression of CD14, CD34, and CD45 (Figure S2C,D).

**FIGURE 3:**
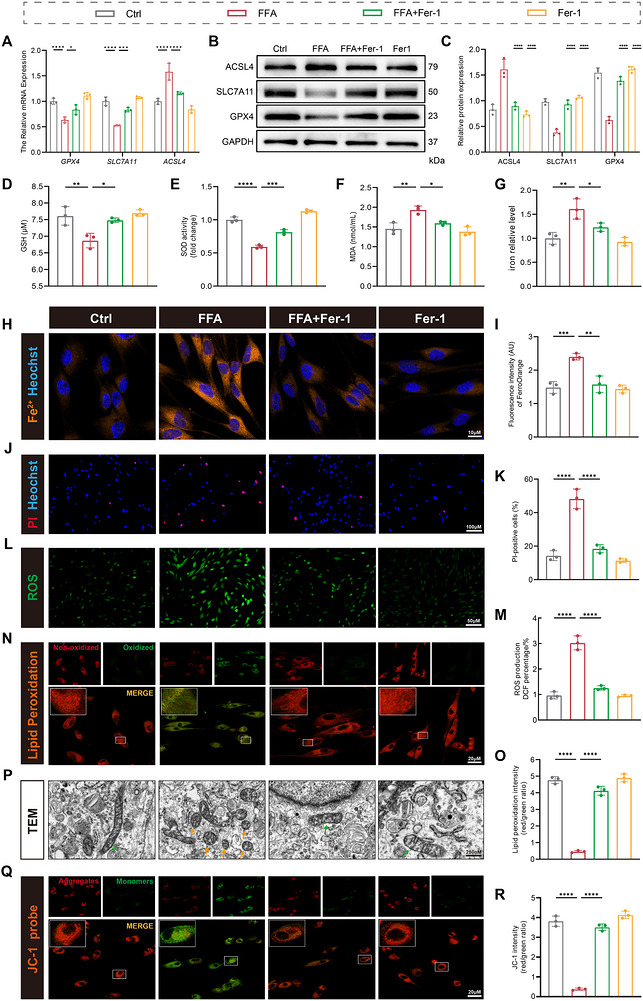
Free fatty acids induce ferroptosis in PDLSCs in vitro. (A) mRNA expression levels of *GPX4*, *SLC7A11*, and *ACSL4* after FFA treatment. (B) Western blot analysis of ferroptosis‐related proteins ACSL4, SLC7A11, and GPX4. (C) Quantification of band intensity for ACSL4, SLC7A11, and GPX4. (D,E) Measurement of intracellular glutathione (GSH) and superoxide dismutase (SOD) levels after FFA treatment. (F) Detection of malondialdehyde (MDA), a lipid peroxidation end‐product. (G) Analysis of intracellular iron (Fe^2^^+^) levels. (H) Fluorescent probe detection of intracellular ferrous iron (Fe^2^^+^) accumulation. (I) Quantification of Fe^2^^+^ fluorescence intensity. (J) PI/Hoechst double staining to evaluate cell death. (K) Quantification of PI‐positive cell percentage. (L) Detection of intracellular reactive oxygen species (ROS) using the DCFH‐DA fluorescent probe. (M) Quantification of DCFH‐DA fluorescence intensity. (N) Lipid ROS detection using BODIPY 581/591 C11 probe; red indicates non‐oxidized lipids, green indicates oxidized lipids. (O) Quantification of lipid peroxidation fluorescence intensity. (P) Transmission electron microscopy (TEM) images showing mitochondrial ultrastructural changes. (Q) JC‐1 staining to assess mitochondrial membrane potential (MMP); red represents JC‐1 aggregates, green indicates monomers. (R) Quantification of red/green fluorescence ratio from JC‐1 staining. (n = 3 per group; values represented mean ± SD; ns indicates no significant, ^*^ *p* < 0.05, ^**^ *p* < 0.01, ^***^ *p* < 0.001, ^****^ *p* < 0.0001).

hPDLSCs were treated with 400 µM free fatty acids (FFA) to establish a hyperlipidemia cell model. Ferrostatin‐1(Fer‐1), a specific ferroptosis inhibitor that blocks lipid peroxidation and protects cells from ferroptotic damage, was used for rescue experiments. CCK‐8 and Western blot analyses confirmed that these concentrations effectively established injury and rescue models (Figure S3A–D). FFA stimulation significantly upregulated the ferroptosis‐related marker ACSL4 at both mRNA and protein levels, while markedly downregulating the antioxidant defense molecules SLC7A11 and GPX4 (Figure 3A–C). Co‐treatment with Fer‐1 reversed these changes, restoring expression to levels comparable to the control group. Moreover, Propidium iodide (PI)/Hoechst double staining revealed a substantial increase in cell death following FFA treatment, which was significantly alleviated by Fer‐1, indicating that FFA induces ferroptosis in hPDLSCs (Figure 3J,K).

Ferroptosis is an iron‐dependent form of programmed cell death characterized by the accumulation of lipid peroxides, typically resulting from the collapse of the cellular antioxidant system [13]. Glutathione (GSH) and superoxide dismutase (SOD) are crucial in maintaining redox homeostasis: GSH serves as a major reducing agent that scavenges ROS, while SOD is a frontline antioxidant enzyme that catalyzes the conversion of superoxide radicals into hydrogen peroxide [25]. In our study, FFA treatment significantly decreased intracellular GSH and SOD levels in hPDLSCs, indicating a compromised antioxidant defense system (Figure 3D,E). Simultaneously, malondialdehyde (MDA), a terminal product of lipid peroxidation, was markedly elevated, reflecting increased lipid oxidative damage and cellular oxidative stress (Figure 3F) [26]. Notably, Fer‐1 co‐treatment effectively restored GSH and SOD levels and reduced MDA accumulation, suggesting that Fer‐1 alleviates FFA‐induced redox imbalance and lipid peroxidation, thereby inhibiting ferroptosis. Beyond the collapse of the antioxidant defense, ferroptosis is also closely linked to disrupted iron metabolism, particularly through the Fenton reaction, in which Fe^3^^+^ is reduced to ferrous iron (Fe^2^^+^), generating highly reactive hydroxyl radicals that exacerbate oxidative injury [27]. Consistent with this mechanism, our results demonstrated a significant increase in intracellular Fe^2^^+^ levels following FFA treatment (Figure 3G–I), further supporting the involvement of iron dysregulation in ferroptosis. To more comprehensively evaluate oxidative stress, we employed DCFH‐DA and BODIPY 581/591 C11 probes to detect total ROS and lipid ROS, respectively, and observed significant elevations after FFA exposure (Figure 3L–O), thereby confirming the occurrence of aggravated intracellular oxidative damage. Importantly, Fer‐1 treatment effectively reversed these alterations by reducing Fe^2^^+^ accumulation and suppressing both total and lipid ROS levels, ultimately restoring redox homeostasis, attenuating lipid peroxidation, and mitigating ferroptosis.

Given the central role of mitochondria in ferroptosis, we then examined mitochondrial ultrastructure using transmission electron microscopy (TEM). In control cells, mitochondria displayed intact membranes, well‐organized cristae, and normal morphology, consistent with healthy mitochondrial function. In contrast, FFA‐treated cells showed typical ferroptotic features, including mitochondrial shrinkage, increased membrane density, and cristae loss. These structural abnormalities were significantly alleviated by co‐treatment with Fer‐1 (Figure 3P). Additionally, JC‐1 staining revealed pronounced mitochondrial membrane potential (MMP) depolarization following FFA treatment, which was effectively reversed by Fer‐1, indicating restored mitochondrial function (Figure 3Q,R).

Moreover, representative markers of apoptosis, necroptosis, and pyroptosis were not markedly activated after FFA treatment in hPDLSCs (Figure S4A–C), further supporting ferroptosis as the predominant mode of cell death under our experimental conditions.

### 2.4 Fer‐1 Restores PDLSC Osteogenesis Under Hyperlipidemia and Attenuates Inflammatory Periodontal Bone Loss

**FIGURE 4:**
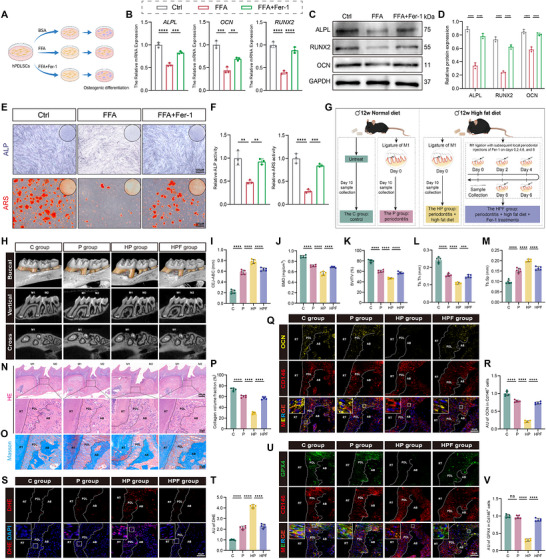
Fer‐1 restores PDLSC osteogenesis under dysregulated lipid metabolism and alleviates periodontal bone loss. (A) Schematic illustration of the experimental design showing osteogenic induction of hPDLSCs treated with BSA (Ctrl), FFA or FFA + Fer‐1. (B) RT‐qPCR analysis of *ALPL*, *OCN*, and *RUNX2* mRNA expression in hPDLSCs treated with FFA or FFA combined with Fer‐1. (C) Western blot analysis of ALPL, RUNX2, and OCN protein levels. (D) Quantification of band intensity for ALPL, RUNX2, and OCN proteins. (E) ALP staining and ARS staining to assess osteogenic potential and mineralized nodule formation. (F) Quantification of ALP activity and mineralized area. (G) Schematic illustration of the in vivo experimental design, including: control group (C), periodontitis group (P), high fat diet with periodontitis group (HP), and high fat diet with Fer‐1 treatment group (HPF). (H) Representative micro‐CT images of alveolar bone in buccal, vertical, and cross‐sectional views across groups. (I–M) Quantitative analysis of alveolar bone structural parameters: CEJ–ABC, BMD, BV/TV, Tb.Th and Tb.Sp. (N) HE staining of periodontal tissue morphology. (O) Masson's trichrome staining to assess collagen deposition in the PDL region. (P) Quantification of collagen area in the PDL. (Q,U) Immunofluorescence co‐staining of CD146 with OCN (Q) or GPX4 (U) to visualize osteogenic marker expression in PDLSCs. (R,V) Quantification of the mean fluorescence intensity of OCN (R) and GPX4 (V) in Cd146^+^ cells. (S) DHE fluorescence staining to detect ROS accumulation in the PDL region. (T) Quantification of DHE fluorescence intensity. (A–G: n = 3 per group; H–V: n = 5 per group; values represented mean ± SD; ns indicates no significant, ^*^ *p* < 0.05, ^**^ *p* < 0.01, ^***^ *p* < 0.001, ^****^ *p* < 0.0001).

To investigate the impact of FFA‐induced ferroptosis on the osteogenic differentiation capacity of hPDLSCs, we first examined the expression of key osteogenic markers using RT‐qPCR and western blotting. The results showed that treatment with FFA significantly downregulated the mRNA and protein levels of *ALPL*, *OCN*, and *RUNX2* (Figure 4A–D). However, ferroptosis inhibitor Fer‐1 partially reversed this suppression, suggesting a protective role against FFA‐induced impairment of osteogenesis. Consistently, alkaline phosphatase (ALP) staining and Alizarin Red S (ARS) staining revealed reduced mineralization and osteogenic potential in the FFA group, while Fer‐1 treatment markedly rescued these defects (Figure 4E,F). These findings indicate that ferroptosis plays a crucial role in mediating FFA‐induced inhibition of hPDLSCs osteogenic differentiation.

To further validate the role of ferroptosis in alveolar bone remodeling under hyperlipidemic and inflammatory conditions, 12‐week‐old male mice were randomly assigned to four groups: Control group (C group), fed a standard chow diet without any treatment; Periodontitis group (P group), subjected to ligature placement around the maxillary first molars (M1) on day 0 to induce periodontitis; High fat diet with periodontitis group (HP group), fed a high fat diet for 12 weeks prior to ligature placement and sacrificed on day 10; and HP group with Fer‐1 treatment group (HPF group), which received the same high fat diet and ligature induction as the HP group, but additionally received local periodontal injections of Fer‐1 on days 0, 2, 4, 6, and 8 (Figure 4G; Figure S5A–F). Mice were sacrificed at day 10 for sample collection.

The results revealed that the HP group exhibited significantly greater alveolar bone resorption compared to the P group, as indicated by increased CEJ–ABC distance (Figure 4H,I). Bone quality metrics, including BMD, BV/TV, and Tb.Th, were markedly reduced in the HP group, while Tb.Sp was significantly elevated. Importantly, Fer‐1 treatment in the HPF group resulted in substantial recovery of vertical bone defects and bone quality parameters (Figure 4J–M). Moreover, histological analysis showed reduced collagen deposition in the periodontal ligament region of the HP group compared to the P group (Figure 4N–P). Immunostaining indicated a decreased expression of OCN in hPDLSCs from the HP group, suggesting impaired osteogenic differentiation capacity under dyslipidemic conditions, further tipping the balance toward bone resorption (Figure 4Q,R). Notably, the HP group also showed elevated ROS accumulation and downregulated GPX4 expression in the PDL region, confirming ferroptosis activation (Figure 4S–V). In contrast, Fer‐1 treatment in the HPF group effectively reduced vertical bone defects, improved bone quality parameters, alleviated ROS accumulation, preserved GPX4 expression, and restored osteogenic potential in hPDLSCs (Figure 4S–V). Taken together, these findings indicate that ferroptosis plays a pivotal role in periodontal bone destruction induced by dysregulated lipid metabolism.

### 2.5 Lipid Metabolism‐Induced Ferroptotic Stress Disrupts the GSK3β/NRF2 Regulatory Axis in PDLSCs

**FIGURE 5:**
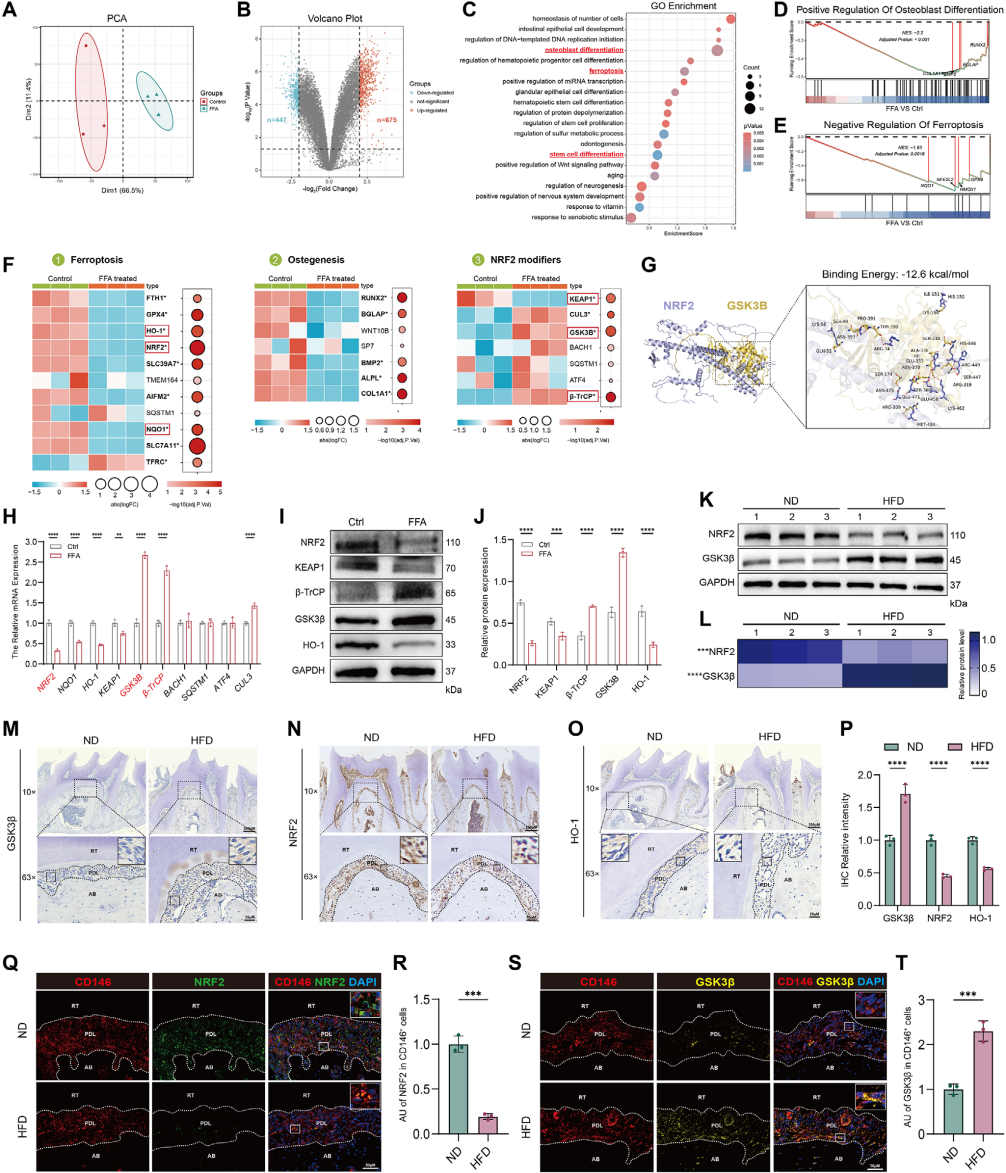
Lipid metabolism‐induced ferroptotic stress disrupts the GSK3β/NRF2 regulatory axis in PDLSCs. (A) Principal component analysis (PCA) illustrating gene expression differences between control and FFA‐treated PDLSCs. (B) Volcano plot showing the distribution of differentially expressed genes (DEGs). (C) GO enrichment analysis of DEGs. (D,E) Gene set enrichment analysis (GSEA) reveals significant enrichment of positively regulated osteogenic differentiation‐related genes (D) and negatively regulated ferroptosis‐related genes (E) in the FFA‐treated group. (F) Heatmaps of genes associated with ferroptosis, osteogenesis, and NRF2 signaling in control and FFA‐treated PDLSCs. (G) Molecular docking simulation demonstrating strong binding affinity between NRF2 and GSK3β. (H) RT‐qPCR analysis of ferroptosis‐related gene expression in control and FFA‐treated PDLSCs. (I,J) Western blot and quantification of NRF2, KEAP1, β‐TrCP, GSK3β, and HO‐1 protein levels in PDLSCs following FFA treatment. (K,L) Western blot (K) and heatmap (L) showing differential expression of NRF2 and GSK3β in periodontal tissues from mice fed a high fat diet versus normal diet. (M–O) Immunohistochemical staining of GSK3β (M), NRF2 (N), and HO‐1 (O) in periodontal tissues from normal and HFD mice. (P) Quantitative analysis of immunohistochemical signal intensities of GSK3β, NRF2, and HO‐1. (Q,R) Immunofluorescence co‐staining of CD146 with NRF2 (Q) and quantification of the mean fluorescence intensity of NRF2 within CD146^+^ cells (R). (S,T) Immunofluorescence co‐staining of CD146 with GSK3β (S) and quantification of the mean fluorescence intensity of GSK3β within CD146^+^ cells (T). (A–L: n = 3 per group; M–T: n = 5 per group; values represented mean ± SD; ns indicates no significant, ^*^ *p* < 0.05, ^**^ *p* < 0.01, ^***^ *p* < 0.001, ^****^ *p* < 0.0001).

To further clarify the mechanism by which abnormal lipid metabolism induces ferroptosis in PDLSCs and reduces their osteogenic differentiation ability, we performed transcriptome sequencing on hPDLSCs treated with 400 µM FFA for 48 h and control cells. The results showed significant differences in gene expression between the two groups: 675 genes were significantly upregulated and 447 genes were significantly downregulated in the FFA group (Figure 5A,B). GO enrichment analysis indicated that the differentially expressed genes were significantly enriched in biological processes such as osteoblast differentiation, stem cell differentiation, and ferroptosis (Figure 5C). Further GSEA analysis showed that, compared with the control group, FFA treatment significantly suppressed key pathways such as osteogenic differentiation and negative regulation of ferroptosis (Figure 5D,E). In the analysis of core regulators of ferroptosis, we found that *NRF2* was significantly downregulated in the FFA group, and its downstream antioxidant‐related genes, including *HO‐1*, *NQO1*, *GPX4*, and *SLC7A11*, were also consistently decreased (Figure 5F). This suggests that the inhibition of NRF2 signaling pathway may lead to reduced ferroptosis resistance in PDLSCs and ultimately affect their osteogenic potential.

To further explore the upstream regulatory mechanisms of NRF2, we scrutinized transcriptome data with RT‐qPCR analysis and found that *GSK3β* and *β‐TrCP* were significantly upregulated in the FFA group, while the classical NRF2 inhibitor *KEAP1* was unexpectedly downregulated. Under physiological conditions, KEAP1 binds NRF2 and promotes its ubiquitin‐mediated degradation, so its downregulation would theoretically provide some protection for NRF2 (Figure 5F,H). Because KEAP1 is a classical negative regulator of NRF2, its downregulation would theoretically be expected to reduce NRF2 degradation and favor NRF2 stabilization. However, NRF2 protein levels were still markedly decreased after FFA treatment, suggesting that KEAP1 is unlikely to be the primary driver of NRF2 loss in this context. We therefore speculate that the reduction in KEAP1 may represent a compensatory feedback response to oxidative stress. Noteworthily, GSK3β can bind to NRF2 and make it more easily recognized by β‐TrCP, leading to KEAP1‐independent degradation and forming an NRF2 regulatory axis independent of KEAP1 [28]. Molecular docking experiments showed a strong binding potential between GSK3β and NRF2, suggesting the possibility of direct regulation (Figure 5G). At the protein level, Western blot results further confirmed the findings: after FFA treatment, GSK3β and β‐TrCP were significantly upregulated, while KEAP1, NRF2, and its downstream target HO‐1 were significantly downregulated, further supporting the dominant role of the GSK3β/NRF2 pathway in NRF2 degradation (Figure 5I,J).

To consolidate the transcription changes in vivo, we analyzed the periodontal ligament tissues of mice fed with a normal diet or high fat diet. Immunohistochemistry and Western blot results showed that in the periodontal ligament tissues of high fat diet mice, NRF2 protein levels were significantly decreased, while GSK3β protein levels were significantly increased (Figure 5K–P). Finally, through immunofluorescence co‐staining of CD146 with NRF2 and GSK3β, we further demonstrated the abnormal expression of the GSK3β/NRF2 pathway in PDLSCs under lipid metabolic disturbance (Figure 5Q–T). These results indicate that under lipid metabolism disorder, the GSK3β/NRF2 axis, as a KEAP1‐independent NRF2 degradation pathway, may play a key role in ferroptosis of PDLSCs and the reduction of their osteogenic potential.

### 2.6 NRF2 Plays a Key Role in Regulating the Susceptibility of PDLSCs to FFA‐Induced Ferroptosis

**FIGURE 6:**
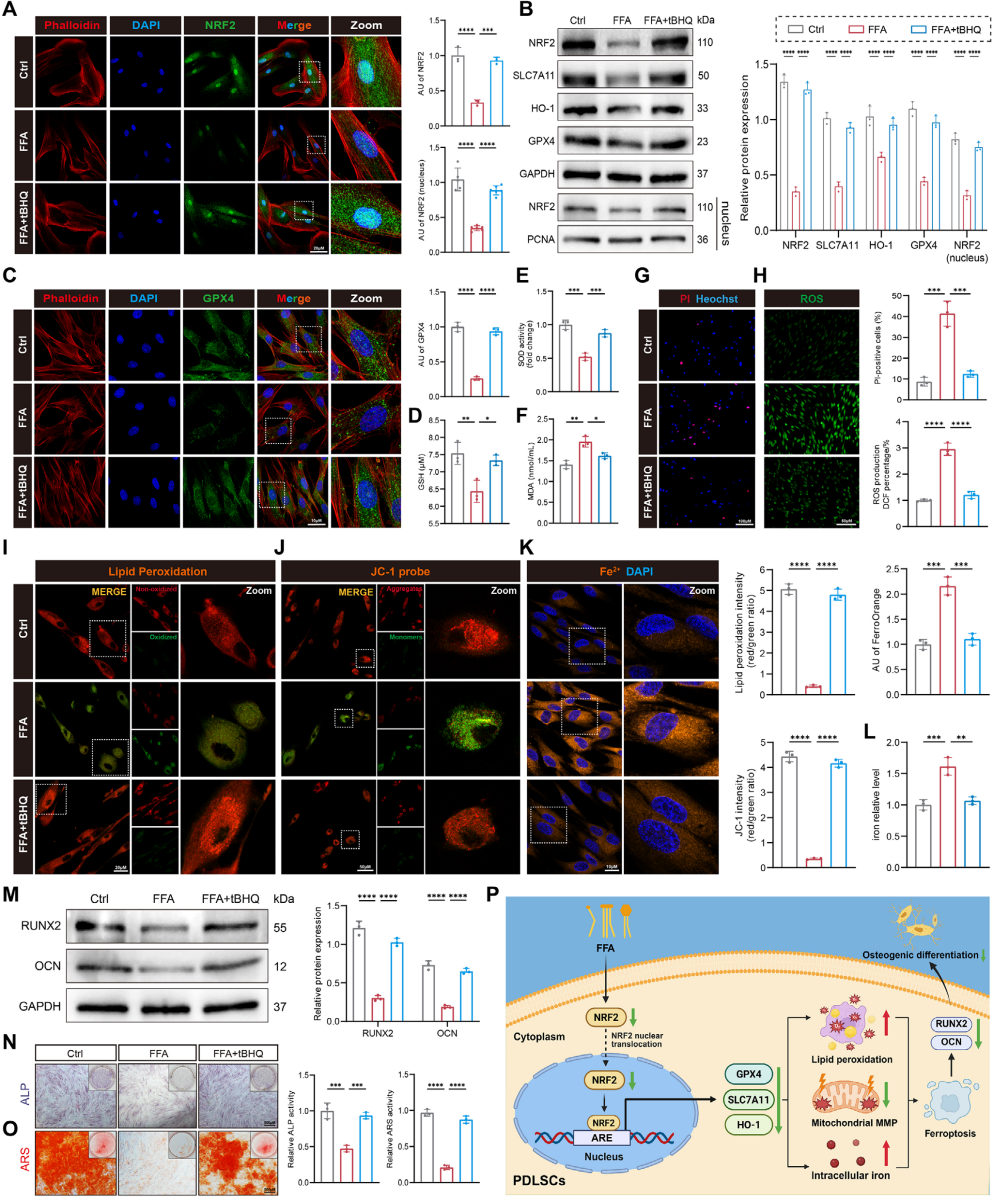
NRF2 plays a key role in regulating the susceptibility of PDLSCs to FFA‐induced ferroptosis. (A) Immunofluorescence staining showing intracellular distribution and nuclear translocation of NRF2 across the three groups. (B) Western blot analysis and quantification of total and nuclear NRF2, along with SLC7A11, HO‐1, and GPX4 protein levels in three groups. (C) Immunofluorescence staining of GPX4 to assess expression differences among the three groups. (D–F) Measurement of GSH (D), SOD (E), and MDA (F) levels in hPDLSCs to evaluate antioxidant capacity and ferroptotic stress. (G) PI/Hoechst double staining to assess cell death among the three groups. (H) ROS levels detected using the DCFH‐DA fluorescent probe. (I) Lipid peroxidation detected using specific probes to assess oxidative damage in hPDLSCs. (J) Mitochondrial membrane potential measured by JC‐1 staining. (K) Intracellular Fe^2^^+^ accumulation detected using a fluorescent Fe^2^^+^ probe. (L) Intracellular ferrous iron (Fe^2^^+^) levels measured using a commercial assay kit in three groups. (M) Western blot analysis of osteogenic markers RUNX2 and OCN. (N,O) ALP and ARS staining of hPDLSCs after osteogenic induction in the three groups. (P) Schematic illustration: FFA‐induced NRF2 degradation triggers ferroptosis in PDLSCs, leading to lipid peroxidation and mitochondrial dysfunction, thereby impairing the osteogenic differentiation capacity of PDLSCs. (n = 3 per group; values represented mean ± SD; ns indicates no significant, ^*^ *p* < 0.05, ^**^ *p* < 0.01, ^***^ *p* < 0.001, ^****^ *p* < 0.0001).

To further validate that NRF2 is a key regulator of ferroptosis susceptibility in PDLSCs under lipid metabolic disturbance, we performed a series of rescue experiments using tBHQ, a specific activator of NRF2. CCK‐8 assays determined that treating hPDLSCs with 20 µM tBHQ for 24 h was optimal (Figure S6A). tBHQ markedly restored the FFA‐induced reduction in both total and nuclear NRF2 protein levels (Figure 6A,B). The reactivation of nuclear translocation of NRF2 facilitated the transcriptional upregulation of antioxidant response elements (AREs), including GPX4, SLC7A11, and HO‐1, as confirmed by elevated mRNA and protein levels (Figure 6B,C; Figure S6B).

Restoration of NRF2 levels led to increased intracellular levels of GSH and SOD, along with a significant reduction in MDA, indicating that tBHQ enhances the antioxidant capacity of hPDLSCs and alleviates oxidative stress (Figure 6D–F). Similarly, we observed that tBHQ treatment attenuated key indicators of ferroptotic responses, including reduced cell death rate (Figure 6G), total ROS levels (Figure 6H), lipid peroxidation (Figure 6I), and intracellular Fe^2^^+^ accumulation (Figure 6K,L), while also restoring impaired mitochondrial membrane potential (Figure 6J). These findings strongly support the notion that NRF2 plays a critical role in regulating the susceptibility of PDLSCs to ferroptosis under lipotoxic conditions.

We also investigated whether tBHQ could rescue the osteogenic differentiation potential of hPDLSCs impaired by FFA exposure. Western blot and RT‐qPCR analyses demonstrated that tBHQ co‐treatment restored the mRNA and protein expression levels of osteogenic markers RUNX2 and OCN (Figure 6M; Figure S6C,D). Moreover, ALP staining and ARS staining revealed that tBHQ significantly improved early osteogenic activity and calcium nodule formation compared to the FFA group alone (Figure 6N,O). These results suggest that tBHQ restores NRF2 activity suppressed by FFA, thereby protecting hPDLSCs from ferroptosis and rescuing their osteogenic differentiation capacity (Figure 6P).

### 2.7 GSK3β Promotes FFA‐Induced Ferroptosis in PDLSCs Through NRF2 Ubiquitin‐Proteasome Degradation

**FIGURE 7:**
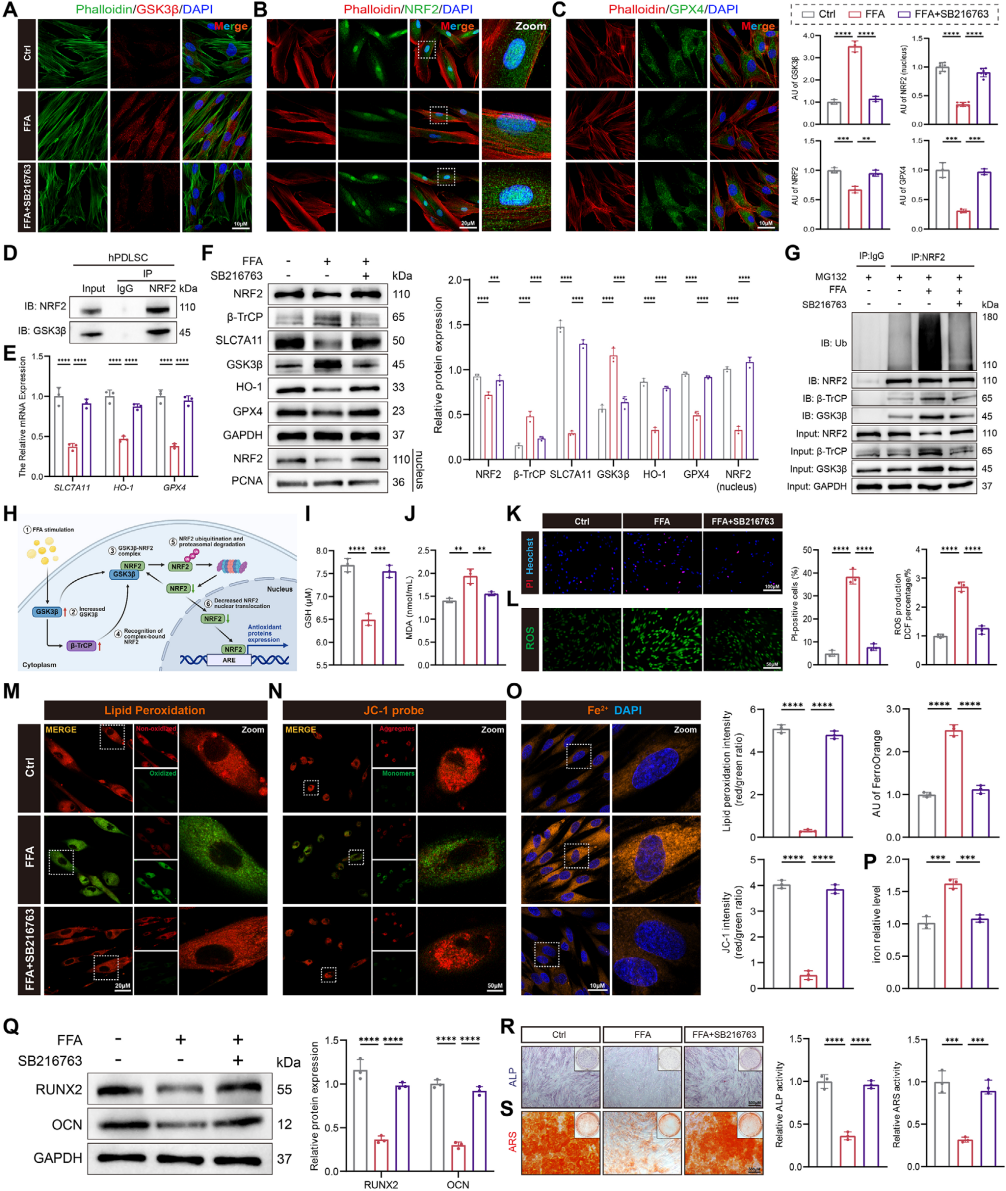
GSK3β promotes FFA‐induced ferroptosis in PDLSCs through NRF2 ubiquitin‐proteasome degradation. (A–C) Immunofluorescence staining of GSK3β (A), NRF2 (B), and GPX4 (C) expression in the three groups. (D) Endogenous co‐immunoprecipitation (Co‐IP) assay showing the interaction between GSK3β and NRF2 in hPDLSCs. (E) RT‐qPCR analysis of SLC7A11, HO‐1, and GPX4 mRNA expression. (F) Western blot analysis of total and nuclear NRF2, β‐TrCP, SLC7A11, GSK3β, HO‐1, and GPX4 in the three groups. (G) Co‐IP assay showing increased NRF2 ubiquitination in FFA‐treated hPDLSCs, which was significantly reduced upon SB216763 treatment. (H) Schematic illustration: FFA disrupts cellular antioxidant defense by promoting GSK3β‐dependent ubiquitination and degradation of NRF2. (I,J) Quantification of intracellular GSH (I) and MDA (J) levels in the three groups. (K) PI/Hoechst double staining to assess cell death rates. (L) DCFH‐DA fluorescent probe to detect intracellular ROS levels. (M) Lipid peroxidation detection probe used to evaluate oxidative lipid damage. (N) JC‐1 staining to assess mitochondrial membrane potential changes. (O) Fe^2^^+^ fluorescent probe for detection and quantification of intracellular ferrous ion accumulation. (P) Intracellular ferrous iron (Fe^2^^+^) levels measured using a commercial assay kit in three groups. (Q)  Western blot and densitometric analysis of the osteogenic markers RUNX2 and OCN. (R,S) ALP and ARS staining to evaluate early osteogenic differentiation and mineralization capacity. (n = 3 per group; values represented mean ± SD; ns indicates no significant, ^*^ *p* < 0.05, ^**^ *p* < 0.01, ^***^ *p* < 0.001, ^****^ *p* < 0.0001).

After identifying NRF2 as the key mediator of FFA‐induced hPDLSCs death, we next aimed to determine whether the upstream regulator of NRF2 in response to lipid metabolic dysfunction is the GSK3β/NRF2 axis identified by transcriptome sequencing. To this end, we conducted rescue experiments using the GSK3β‐specific inhibitor SB216763. CCK8 assays confirmed that 10 µM SB216763 for 24 h is an appropriate concentration for treating hPDLSCs (Figure S8A). Immunofluorescence and Western blot analyses revealed that SB216763 reversed the elevated GSK3β levels induced by FFA in hPDLSCs (Figure 7A,F). Following the suppression of GSK3β, both total and nuclear NRF2 levels were restored, confirming that GSK3β upregulation plays a central role in NRF2 suppression (Figure 7B). The subsequent recovery of NRF2 nuclear translocation upregulated the mRNA and protein levels of ARE‐dependent antioxidant molecules, including GPX4, SLC7A11, and HO‐1 (Figure 7C,E,F). Endogenous immunoprecipitation (IP) assays in hPDLSCs demonstrated a direct interaction between GSK3β and NRF2 (Figure 7D). Notably, inhibition of GSK3β led to a marked reduction in β‐TrCP protein expression, and weakened the association between GSK3β and NRF2, indicating that β‐TrCP functions as a downstream effector of GSK3β in promoting NRF2 degradation (Figure 7F). Co‐immunoprecipitation assays showed that FFA treatment increased NRF2 ubiquitination in hPDLSCs, accompanied by enhanced binding of β‐TrCP to NRF2, which was reduced upon SB216763 treatment (Figure 7G). To further investigate the role of β‐TrCP in NRF2 degradation, β‐TrCP was silenced in FFA‐treated hPDLSCs. β‐TrCP silencing markedly restored total and nuclear NRF2 protein levels and rescued the expression of the NRF2 downstream antioxidant proteins SLC7A11, HO‐1, and GPX4 (Figure S7A). In addition, Co‐IP analysis showed that knockdown of β‐TrCP reduced its association with NRF2 and attenuated NRF2 ubiquitination, although GSK3β expression and its interaction with NRF2 remained elevated compared with the control group (Figure S7B). This finding supports the following mechanism: FFA‐induced upregulation of GSK3β in hPDLSCs enhances its interaction with NRF2, making NRF2 more susceptible to recognition and ubiquitination by β‐TrCP, followed by proteasomal degradation. Meanwhile, elevated GSK3β also increases the expression of β‐TrCP itself, forming a positive feedback loop that further amplifies NRF2 degradation (Figure 7H).

We also investigated whether GSK3β upregulation contributes to FFA‐induced ferroptosis and impaired osteogenic differentiation in hPDLSCs. Redox assays indicated that SB216763 treatment restored intracellular antioxidant capacity, as evidenced by increased GSH and SOD levels and decreased MDA content (Figure 7I,J; Figure S8B). Ferroptosis‐related phenotypes, including cell death rate (Figure 7K), total ROS (Figure 7L), lipid peroxidation (Figure 7M), and intracellular ferrous iron levels (Figure 7O,P), were significantly reduced following SB216763 treatment, accompanied by a recovery of mitochondrial membrane potential (Figure 7N). Osteogenic markers RUNX2 and OCN, which were suppressed by FFA, were restored by SB216763 (Figure 7Q; Figure S8C,D). ALP and Alizarin Red staining further confirmed that early osteogenic differentiation and calcium nodule formation capacities were rescued by GSK3β inhibition (Figure 7R,S). Collectively, these results demonstrate that NRF2 mediates FFA‐induced ferroptosis in PDLSCs through a GSK3β‐dependent ubiquitin‐proteasome degradation pathway.

### 2.8 SB216763 Alleviates PDLSCs Ferroptosis and Alveolar Bone Loss in Hyperlipidemia‐Associated Periodontitis Mice by Modulating the GSK3β/NRF2 Axis

**FIGURE 8:**
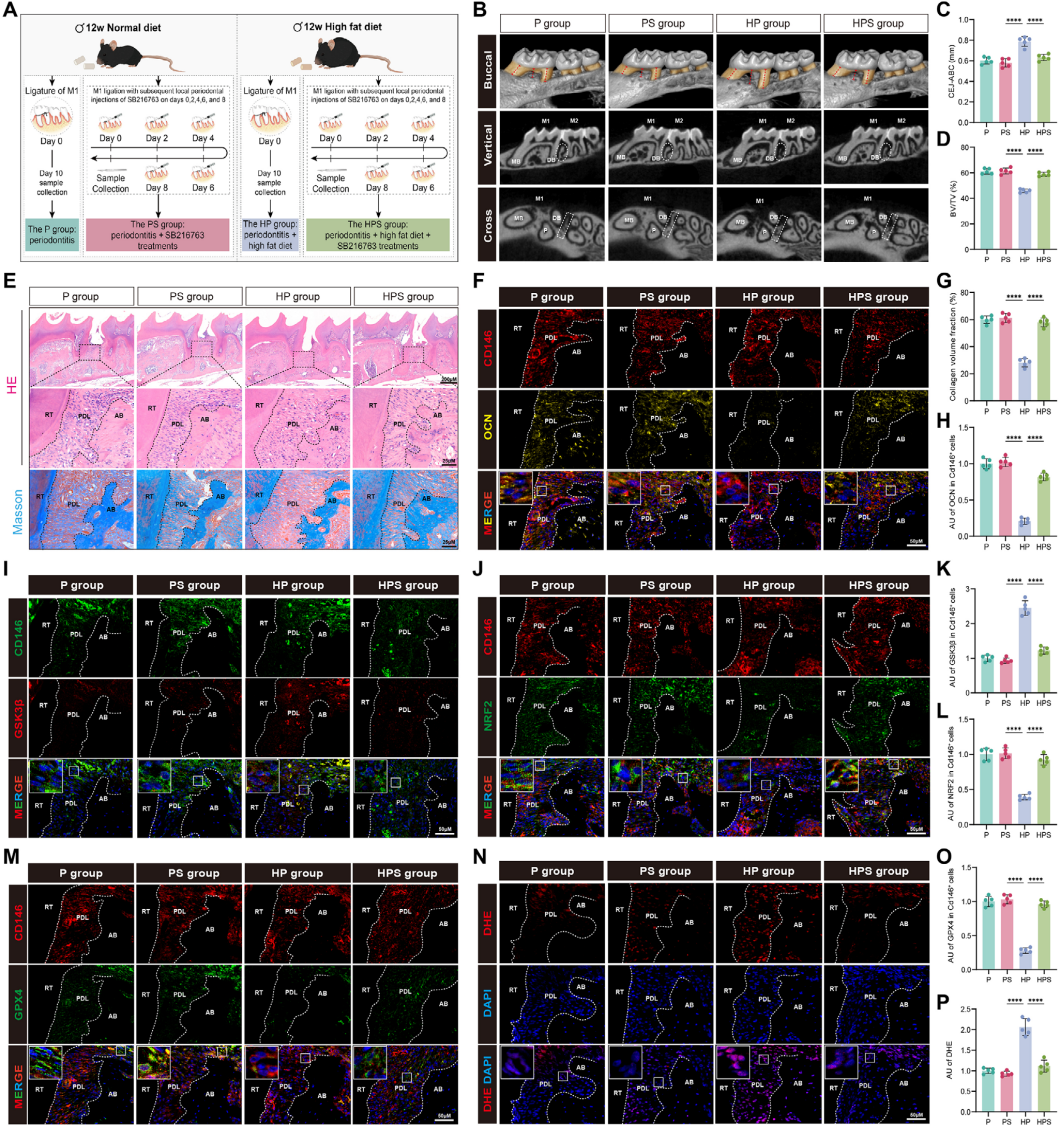
SB216763 alleviates ferroptosis of PDLSCs and alveolar bone loss in lipid metabolism‐related periodontitis mice by modulating the GSK3β/NRF2/GPX4 pathway. (A) Schematic illustration of the animal experimental design. (B) Representative micro‐CT images of alveolar bone in each group, shown in buccal, vertical, and cross‐sectional views. (C,D) Quantitative analysis of CEJ–ABC distance and BV/TV based on micro‐CT images. (E) H&E and Masson's trichrome staining of periodontal tissues from each group. (F) Immunofluorescence co‐staining of CD146 and OCN in the PDL region. (G) Quantification of collagen content in the PDL area. (H) Quantification of the mean fluorescence intensity of OCN within CD146^+^ cells. (I,J) Immunofluorescence co‐staining of CD146 with GSK3β (I) and NRF2 (J) in the PDL region. (K,L)  Quantification of the mean fluorescence intensity of GSK3β (K) and NRF2 (L) within CD146^+^ cells. M) Immunofluorescence co‐staining of CD146 and GPX4 in the PDL region. (N) DHE fluorescence staining to detect ROS levels in PDL. (O)  Quantification of the mean fluorescence intensity of GPX4 within CD146^+^ cells. (P) Quantification of DHE fluorescence intensity. (n = 5 per group; values represented mean ± SD; ns indicates no significant, ^*^ *p* < 0.05, ^**^ *p* < 0.01, ^***^ *p* < 0.001, ^****^ *p* < 0.0001).

To further validate in vivo the role of the GSK3β/NRF2 axis in promoting PDLSCs ferroptosis and exacerbating periodontal bone loss under hyperlipidemic condition, we designed a rescue animal experiment using specific GSK3β inhibitor SB216763. The mice were divided into four groups: periodontitis with normal diet (P group), periodontitis with normal diet plus SB216763 intervention (PS group), periodontitis with high fat diet (HP group), and periodontitis with high fat diet plus SB216763 intervention (HPS group). Results showed that mice in the HP and HPS groups exhibited clear signs of hyperlipidemia compared to the P and PS groups (Figure 8A; Figure S9A–F). Micro‐CT analysis revealed that CEJ–ABC distance was significantly reduced in the HPS group compared to the HP group (Figure 8B,C). Additionally, alveolar bone parameters, including BV/TV and Tb.Th, were significantly increased, while Tb.Sp was reduced in the HPS group, indicating that SB216763 effectively alleviated the progression of alveolar bone loss in hyperlipidemic periodontitis mice (Figure 8D; Figure S10A,B). Bone height and quality in the HPS group were restored to levels comparable to the normolipidemic periodontitis group. Notably, no significant differences in alveolar bone loss or bone quality were observed between the P and PS groups, suggesting that SB216763 has no therapeutic effect on periodontitis under normal lipid metabolism.

To elucidate the underlying mechanisms by which SB216763 alleviates inflammatory alveolar bone loss under hyperlipidemic condition, we performed Masson's trichrome staining of periodontal ligament tissue. The results showed restored collagen deposition in the HPS group (Figure 8E,G). Furthermore, osteogenic markers OCN and RUNX2, which were reduced in the HP group compared to the P group, were markedly upregulated in the HPS group, indicating that local administration of SB216763 restored the osteogenic potential of PDLSCs in hyperlipidemic conditions (Figure 8F,H; Figure S10C).

Mechanistically, SB216763 treatment in the HPS group reduced GSK3β expression in the periodontal ligament (Figure 8I,K), which led to decreased NRF2 degradation and elevated NRF2 levels (Figure 8J,L), subsequently restoring the expression of the ferroptosis‐inhibitory protein GPX4 (Figure 8M,O) and suppressing ferroptosis. DHE staining further revealed a significant reduction in total ROS levels in the HPS group compared to the HP group (Figure 8N,P). Collectively, these findings demonstrate that local SB216763 administration confers a protective effect against hyperlipidemia‐induced PDLSC ferroptosis by modulating the GSK3β/NRF2/GPX4 axis, thereby restoring osteogenic differentiation capacity and attenuating alveolar bone loss in hyperlipidemia‐associated periodontitis.

## 3 Discussion

Our findings provide evidence for a previously uncharacterized mechanism linking hyperlipidemia to periodontal tissue destruction through ferroptosis of PDLSCs. Dyslipidemia, particularly hyperlipidemia, is a well‐established systemic risk factor known to exacerbate periodontal inflammation and accelerate alveolar bone loss [29, 30]. Building upon this knowledge, our study demonstrates that excessive lipid accumulation promotes GSK3β‐mediated ubiquitin‐proteasome degradation of NRF2, thereby triggering oxidative stress‐induced ferroptosis in PDLSCs. While some components of this regulatory framework have been investigated in other settings, our study extends these observations to the periodontal microenvironment and identifies PDLSCs as a previously underappreciated cellular target of lipotoxic ferroptotic injury. Given the essential role of PDLSCs in periodontal homeostasis and alveolar bone regeneration, this mechanism advances the current pathophysiological paradigm of periodontitis under metabolic disorders by suggesting that dyslipidemia impairs periodontal regenerative capacity not only through generalized oxidative damage, but also through direct disruption of stem cell function. Furthermore, our data suggest that, under hyperlipidemic stress, NRF2 suppression in PDLSCs is associated with GSK3β/β‐TrCP‐mediated, KEAP1‐independent ubiquitin‐proteasome degradation, highlighting a stem cell‐centered and potentially therapeutically relevant mechanism linking dyslipidemia to alveolar bone resorption in the context of periodontitis.

NRF2 is a central transcription factor responsible for maintaining redox homeostasis and promoting cell survival under stress conditions [31]. Under physiological circumstances, NRF2 activity is tightly regulated by negative modulators such as KEAP1 and β‐TrCP [32]. The KEAP1–NRF2 axis, the most extensively studied pathway, involves KEAP1 binding to NRF2 at both ETGE and DLG motifs, linking NRF2 to the CUL3–RBX1 E3 ubiquitin ligase complex and promoting its proteasomal degradation—a process implicated in the pathogenesis of Parkinson's disease, Alzheimer's disease, stroke, and chronic kidney disease [33, 34, 35, 36]. More recently, GSK3β has emerged as an alternative regulator of NRF2 degradation through its interaction with NRF2, thereby facilitating KEAP1‐independent, β‐TrCP‐mediated ubiquitination [37]. Our results indicate that dyslipidemia enhances GSK3β‐driven NRF2 degradation in PDLSCs, weakening the cytoprotective antioxidant response of NRF2. Notably, although dyslipidemia reduced KEAP1 expression, NRF2 levels were still markedly decreased, indicating that KEAP1 is unlikely to be the dominant driver of NRF2 degradation in this setting. Instead, the reduction in KEAP1 may reflect a compensatory response, whereas GSK3β/β‐TrCP‐mediated KEAP1‐independent degradation appears to predominate. Furthermore, we observed significant suppression of NRF2 downstream defenses (e.g., GPX4, SLC7A11, HO‐1) under dyslipidemic conditions, accompanied by the accumulation of lipid peroxides and iron‐biochemical hallmarks of ferroptosis. These findings link the GSK3β–NRF2 axis to PDLSC viability under lipid overload, offering new insights into the osteogenic differentiation mechanisms of PDLSCs under metabolic stress.

Given the pleiotropic nature of GSK3β, the protective effects of SB216763 should not be interpreted solely through the NRF2 axis. As a key component of the canonical Wnt/β‐catenin pathway, GSK3β inhibition may also promote osteogenesis through β‐catenin stabilization [38]. However, in the present study, our data more strongly support a predominant role of the GSK3β–NRF2–ferroptosis axis. SB216763 restored NRF2 expression and nuclear localization, reduced NRF2 ubiquitination, increased GPX4, SLC7A11, and HO‐1 expression, and alleviated multiple ferroptosis‐related phenotypes under lipotoxic conditions. Moreover, in vivo, the PS group did not show an obvious protective effect against alveolar bone resorption compared with the P group, whereas the HPS group exhibited significantly delayed periodontal bone loss. These findings suggest that correction of the pathological GSK3β–NRF2–ferroptosis axis may play a more important role under hyperlipidemic conditions, while activation of Wnt/β‐catenin signaling may still contribute in a cooperative manner. Future studies combining direct evaluation of β‐catenin activity or pathway‐specific genetic and pharmacological approaches are needed to further dissect the relative contributions of the NRF2/ferroptosis axis and Wnt‐mediated osteogenesis.

Hyperlipidemia‐associated GSK3β activation may arise from multiple upstream inputs, and at least two non‐mutually exclusive mechanisms should be considered. First, dyslipidemia is often accompanied by systemic low‐grade inflammation, which may increase pro‐inflammatory cytokine signaling, such as TNF‐α and IL‐1β, in periodontal tissues [39, 40, 41]. In this setting, interaction with the PI3K–Akt pathway is particularly relevant because Akt‐mediated Ser9 phosphorylation is a key inhibitory checkpoint for GSK3β [40]. Therefore, inflammatory or metabolic stress that weakens Akt signaling may favor a more active GSK3β state [39]. Second, hyperlipidemia also increases exposure to lipotoxic metabolites, especially saturated fatty acids and ceramides, which have been implicated in impaired insulin/Akt signaling [42, 43]. For instance, palmitate has been associated with cellular insulin resistance and altered Akt downstream signaling. In parallel, ceramides may trigger PP2A activation and promote Akt dephosphorylation, which could further release GSK3β from inhibitory control [42]. Taken together, current evidence supports a working model in which inflammatory cytokine inputs and lipid‐derived metabolites converge on Akt/PP2A‐related signaling nodes to promote GSK3β activation. However, the relative contribution of these pathways in PDLSCs within the periodontal niche under hyperlipidemic conditions remains to be clarified.

Importantly, our identification of ferroptosis in PDLSCs under hyperlipidemic conditions offers a novel perspective on periodontal degeneration in patients with systemic lipid disorders. Prior studies on oxidative stress in periodontitis have focused primarily on inflammation‐induced osteoclast activation and bone resorption [44, 45]. Our data highlight a parallel mechanism whereby impaired antioxidant defenses render osteogenic and reparative cells—such as PDLSCs—susceptible to intrinsic cell death, contributing to tissue breakdown. This aligns with emerging evidence from related fields. For instance, Tang et al. recently demonstrated that ferroptosis occurs in osteoblasts and osteocytes during periodontitis, acting as a catalyst for bone loss by enhancing osteoclastogenesis and inhibiting bone formation [46]. Induced ferroptosis exacerbated inflammation and bone resorption, whereas its inhibition with Liproxstatin‐1 protected against alveolar bone loss [47]. Another study using a periapical periodontitis model showed that ferroptotic macrophages drove periapical bone destruction, associated with NRF2 downregulation and ROS overproduction—effects reversed by NRF2 overexpression or ferroptosis inhibition [48]. These studies support our central premise that insufficient NRF2‐mediated antioxidant activity can trigger periodontal ferroptosis, exacerbating bone degradation.

Our work further underscores the upstream role of metabolic disturbances—specifically dyslipidemia—in this process. Previous reports have shown that chronic hyperlipidemia and hypercholesterolemia induce systemic oxidative stress and low‐grade inflammation, potentially impairing PDLSC function [49, 50, 51]. Beyond lipid abnormalities, metabolic adaptations associated with obesity, including insulin resistance, have emerged as important modifiers of inflammatory and redox signaling [52]. For example, recent studies suggest that insulin resistance itself may contribute to the exacerbation of periodontal inflammation by disrupting normal host responses and amplifying pro‐inflammatory cytokine networks independent of hyperglycemia [53]. As obese rodents with insulin resistance exhibit worse periodontal outcomes than their metabolically healthier counterparts, this supports the concept that insulin action influences periodontal tissue resilience to metabolic stress [54]. Nevertheless, the relative contribution of insulin resistance and dyslipidemia to periodontal ferroptotic susceptibility remains to be further delineated, and future studies integrating comprehensive metabolic profiling will help clarify their respective roles in modulating periodontal redox homeostasis.

In the context of dyslipidemia, we provide direct evidence that elevated circulating lipids enhance GSK3β activity in PDLSCs, promoting ubiquitination and degradation of NRF2. Mechanistically, this aligns with known signaling pathways: elevated pro‐inflammatory cytokines (e.g., TNF‐α) in dyslipidemia activate GSK3β, while insufficient counter‐regulatory signals (e.g., insulin/Akt activity) fail to suppress it, allowing persistent NRF2 targeting [55]. Consequently, PDLSCs are unable to mount effective antioxidant responses to lipid peroxidation products. Under such stress, PDLSCs may undergo ferroptosis instead of differentiating into osteoblasts or periodontal fibroblasts, thus impairing regeneration and tipping the balance toward net tissue loss. Supporting this hypothesis, recent studies have shown that NRF2 activation via exogenous agents protects PDLSCs from inflammatory damage. For example, Chen et al. found that ALDH2‐induced NRF2 activation blocked ferroptosis in PDLSCs, alleviating inflammation and promoting osteogenic differentiation [56]. Conversely, NRF2 inhibition rendered PDLSCs vulnerable to ferroptosis and impaired their differentiation, mirroring our observations under lipid‐induced stress [56]. Altogether, these findings position NRF2 as a critical defender of periodontal cell viability and function, with GSK3β acting as a potent antagonist when aberrantly activated in metabolic syndromes.

Taken together, these findings suggest that modulation of ferroptosis and the GSK3β–NRF2 axis may be particularly relevant for early intervention aimed at preventing periodontitis progression and delaying alveolar bone loss in the setting of hyperlipidemia. By preserving NRF2‐dependent antioxidant defense, limiting lipid peroxidation‐associated ferroptotic injury, and maintaining the osteogenic and regenerative competence of PDLSCs, such interventions may help sustain the periodontal stem cell niche under metabolic stress and thereby attenuate progressive periodontal destruction. However, reversing established periodontal damage is biologically more demanding than preventing its progression. Once structural breakdown has occurred, effective repair would require not only suppression of ongoing oxidative stress, inflammatory injury, and osteoclastogenic activity, but also restoration of a regenerative microenvironment that supports PDLSC survival, osteogenic commitment, and coordinated reconstruction of the periodontal ligament–cementum–alveolar bone complex [57]. This challenge is supported by previous studies showing that inflammatory microenvironments directly impair the osteogenic differentiation capacity of PDLSCs, while high fat diet‐associated metabolic disturbance can delay bone regeneration and compromise bone quality, thereby creating conditions that are unfavorable for true tissue reconstruction [57, 58]. Moreover, although stem cell‐based approaches have shown promise in periodontal regeneration, current clinical evidence remains limited and heterogeneous, indicating that rebuilding already‐damaged periodontal structures is inherently more complex than preventing their deterioration [59]. Therefore, our findings may be particularly meaningful in the context of early intervention and progression control in hyperlipidemia‐associated periodontitis. At the same time, these observations should not be interpreted as evidence that targeting this pathway alone is sufficient to completely halt disease progression or reverse established periodontal damage.

Clinically, the GSK3β–NRF2–ferroptosis axis holds significant implications for patients with dyslipidemia‐related periodontitis. It suggests that these individuals may experience more severe periodontal destruction, not solely due to heightened inflammation, but also due to compromised local regenerative capacity. Our in vivo findings and the previous epidemiological data reveal that hyperlipidemic mice exhibit greater alveolar bone loss, and individuals with metabolic syndrome show poorer periodontal health [29]. Our study provides mechanistic insight into this phenomenon, emphasizing that restoring redox homeostasis may be particularly beneficial for this population. Among potential therapeutic strategies, NRF2 activation stands out as promising. Studies have shown that both local and systemic NRF2 activation can protect against bone loss in inflammatory models [60, 61, 62]. For instance, the electrophilic compound 4‐octyl itaconate (4‐OI) significantly reduced oxidative damage and bone resorption in experimental periodontitis, effects lost in Nrf2‐knockout mice [60]. Natural antioxidants such as sulforaphane and quercetin also mitigate alveolar bone loss by enhancing NRF2 activity [63, 64]. Furthermore, ferroptosis‐related biomarkers hold potential as indicators of disease activity and treatment response. Elevated levels of malondialdehyde, 4‐hydroxynonenal, and ferroptosis‐inhibitory proteins (e.g., NRF2, GPX4, FSP1) in gingival crevicular fluid may reflect local oxidative stress. Recent studies in diabetic periodontitis have shown that GPX4/SLC7A11 downregulation correlates with increased oxidative damage, reversible by antioxidant therapy [65]. It is thus plausible that patients with poorly controlled lipid profiles may exhibit a similar high‐ferroptosis, low‐NRF2 phenotype. Lifestyle interventions, systemic medications, or adjunctive antioxidant treatments aimed at modulating this imbalance may improve periodontal outcomes. Notably, statins have been reported to enhance periodontal health in hyperlipidemic patients, potentially via NRF2 activation and oxidative stress reduction [66, 67]. While further mechanistic clarification is needed, these findings support the necessity of interdisciplinary approaches to managing periodontitis in metabolic disease contexts.

Notably, the histological regions selected for analysis differed between the non‐ligature hyperlipidemia model and the ligature‐induced periodontitis model. In the non‐ligature model, we focused on the furcation area because micro‐CT analysis revealed more pronounced alveolar bone changes in this region. In contrast, in the ligature‐induced periodontitis model, the furcation area typically exhibited marked local tissue destruction, which limited reliable evaluation of PDL architecture. Therefore, we preferentially analyzed the middle root region, where the PDL remained relatively well preserved. In addition, in the physiological hyperlipidemic mouse model, changes in alveolar bone mass and PDL structure in the middle root region may have been less pronounced than those in the furcation area. This regional difference may be related to variations in biomechanical loading, local bone remodeling activity, and the spatial characteristics of early periodontal tissue responses. Further studies are needed to elucidate the mechanisms by which dyslipidemia contributes to this spatially distinct pattern.

Despite the strengths of our study—including the integration of in vitro PDLSC models and in vivo alveolar bone loss assessments—some limitations must be acknowledged. First, to ensure the most representative and structurally interpretable histological assessment under each experimental condition, different regions of interest were selected in the physiological and pathological (ligature‐induced periodontitis) models. Nevertheless, this model‐specific regional selection may still represent a potential limitation when interpreting regional differences between the two models. Second, our investigation focused on a single mechanistic pathway (GSK3β–NRF2) within the complex network linking dyslipidemia and periodontitis. Other mechanisms, such as the activation of nuclear receptors (e.g., RORα) by cholesterol metabolites, may independently drive inflammation in gingival tissues [29]. Our emphasis on ferroptosis does not exclude these parallel pathways. Third, although our in vivo experiments included both physiological and ligature‐induced periodontitis models, the in vitro experiments were primarily designed to model the lipotoxic microenvironment using FFA treatment without additional inflammatory stimulation. While this design allowed us to focus on the direct effects of lipid stress on PDLSC osteogenic differentiation and the underlying mechanism, it does not fully recapitulate the complex inflammatory microenvironment of periodontitis. Thus, the mechanistic link to the aggravation of periodontitis remains relatively indirect in the current in vitro setting. Future studies should further investigate the potential crosstalk between lipid stress and inflammatory signaling in regulating PDLSC fate and function under periodontitis‐related conditions. Fourth, dyslipidemia in this study was modeled using animal systems and in vitro PDLSC cultures, which may not fully reflect the chronic and systemic characteristics of human hyperlipidemia. Therefore, validation in clinical specimens will be necessary to establish the translational relevance of our findings. Specifically, assessing NRF2 suppression and ferroptosis‐related markers in periodontal tissues from patients with hyperlipidemia, together with direct evaluation of the GSK3β–NRF2–ferroptosis signaling axis, would clarify whether the molecular alterations observed in experimental models are recapitulated in the clinical setting. Furthermore, correlating GSK3β activation status, NRF2 expression levels, and lipid peroxidation markers with clinical parameters of periodontal destruction would further strengthen the pathophysiological basis and clinical significance of our findings. Fifth, although we identified GSK3β as a proximal mediator of NRF2 degradation, the upstream signals triggering its activation under dyslipidemic conditions remain unclear. Whether lipid‐driven GSK3β activation results from direct effects of lipotoxic metabolites on PDLSCs or indirect effects via inflammatory cytokines needs further investigation. Clarifying this distinction could inform more precise therapeutic interventions. Another point that warrants further investigation is whether targeting ferroptosis or the GSK3β–NRF2 axis may also be beneficial after periodontal destruction has already been established under hyperlipidemic conditions. In the present study, Fer‐1 and SB216763 were administered at the time of ligature placement, and the observed effects therefore mainly reflect intervention during disease initiation and progression. Whether such strategies may facilitate recovery or promote repair after alveolar bone resorption has already occurred remains to be clarified. In future studies, we will investigate whether modulation of ferroptosis or the GSK3β–NRF2 pathway can reverse established alveolar bone loss, as well as whether such interventions may exert synergistic effects when combined with conventional periodontal therapies. We also aim to further elucidate the upstream mechanisms responsible for dyslipidemia‐induced GSK3β activation and to validate our findings in clinical cohorts.

In conclusion, this study provides novel evidence that dyslipidemia promotes ferroptosis in PDLSCs by suppressing NRF2 in a GSK3β‐dependent manner, thereby exacerbating oxidative damage and alveolar bone loss. By integrating metabolic stress and regulated cell death into the pathogenic framework of periodontitis, our findings highlight the central role of the NRF2 pathway in defending against ferroptosis and inflammatory bone destruction. This mechanistic insight carries important clinical implications, suggesting that antioxidant therapies or ferroptosis‐targeted interventions—particularly those aimed at restoring the GSK3β–NRF2 axis—may improve periodontal outcomes under hyperlipidemic conditions. Specifically, reactivating NRF2 signaling could enhance the osteogenic potential of PDLSCs, offering a promising therapeutic strategy for patients with severe periodontitis complicated by metabolic disorders.

## 4 Materials and Methods

### 4.1 Animal Experiments

9‐week‐old male C57BL/6J mice were randomly assigned to experimental groups. Each group included five mice (n = 5), a sample size selected based on commonly adopted group sizes in recent murine ligature‐induced periodontitis studies employing micro‐CT‐based quantification of alveolar bone loss, in which n = 5 per group was sufficient to detect significant periodontal destruction [68, 69]. Mice were housed under specific pathogen‐free (SPF) conditions (22 ± 2°C, 55 ± 5% humidity, 12‐h light/dark cycle) with ad libitum access to food and water. Mice in the HP, HPF, and HPS groups were fed a high fat diet containing 60% fat (Research Diets, USA) for 12 weeks. Periodontitis was induced by ligating the maxillary first molar with a 6‐0 silk ligature for 10 days under anesthesia using 1% sodium pentobarbital administered intraperitoneally. Mice were monitored daily to ensure ligature retention, and animals in which the ligature was lost during the experimental period were excluded from the final analysis, in accordance with established ligature‐induced periodontitis protocols [68]. In the intervention groups, Ferrostatin‐1 (2 µM, MCE, USA) or SB216763 (10 µM, MCE, USA) was locally injected into the buccal and palatal gingiva every other day starting from the day of ligation. After 10 days, the mice were euthanized, and the specimens were fixed in 4% paraformaldehyde for 24 h.

Serum samples were obtained via retroorbital bleeding following a 6‐h fasting period. Serum triglycerides (TG), total cholesterol (CHO), low‐density lipoprotein cholesterol (LDL‐C), high‐density lipoprotein cholesterol (HDL‐C), and fasting blood glucose (FBG) levels were quantified using commercial assay kits (Njjcbio, China) according to the manufacturer's instructions. Body weight was recorded, and Lee's index was calculated using the formula: [body weight (g)^1^/^3^ × 1000] / naso‐anal length (cm) to assess obesity‐related metabolic changes.

Hyperlipidemia was operationally defined as a significant elevation in serum TG, CHO, and LDL‐C levels accompanied by a reduction in HDL‐C in mice fed a high fat diet for 12 weeks, relative to age‐matched chow‐fed controls. To distinguish dyslipidemia from overt diabetes, fasting blood glucose levels were evaluated concurrently. All HFD mice exhibited FBG values below 11.1 mmol/L, the widely accepted diagnostic threshold for experimental diabetes in murine models. Collectively, these criteria confirmed that the metabolic phenotype induced by the high fat diet predominantly represented hyperlipidemia rather than diabetic hyperglycemia.

All animal procedures were approved by the Animal Ethics Committee of Tongji Hospital, Tongji Medical College, Huazhong University of Science and Technology (Approval Number: T1‐202406094).

### 4.2 Micro‐CT Analysis

Alveolar bone architecture was evaluated using a high‐resolution micro‐computed tomography system (Skyscan 1176, Bruker, Belgium). Fixed maxillary specimens were scanned at 50 kV and 500 µA with a 1.0‐mm aluminum (Al) filter. Images were acquired at an isotropic voxel size of 9 µm. Reconstruction was performed using NRecon software (Bruker) with identical parameters applied to all samples.

Three‐dimensional analyses were conducted using CTAn software (Bruker). For mice under physiological conditions (ND and HFD groups), the region of interest (ROI) was defined as the alveolar bone within the furcation area of the maxillary first molar. For ligature‐induced periodontitis groups (C, P, HP, HPF, PS, and HPS groups), the ROI was defined as the interproximal alveolar bone between the maxillary first and second molars, corresponding to the site of ligature placement and inflammation. Alveolar bone loss was quantified by measuring the linear distance from the cemento‐enamel junction (CEJ) to the alveolar bone crest (ABC) at standardized anatomical landmarks. All measurements were performed in a blinded manner to ensure analytical consistency.

### 4.3 Tissue Histological Staining Analysis

Mouse maxillae were immersed in 4% neutral buffered formalin for 24 h at room temperature for fixation, followed by decalcification in 10% ethylenediaminetetraacetic acid (EDTA, pH 7.4; Servicebio, China) for 4 weeks, with decalcifying solution refreshed every 2 days. After complete decalcification, the tissues were trimmed, dehydrated, paraffin‐embedded, and sectioned at 5 µm thickness. For general morphological assessment, hematoxylin and eosin (H&E) staining was performed according to standard protocols. Collagen fiber deposition was evaluated using a Masson's trichrome staining kit (Servicebio, China).

For immunohistochemical (IHC) analysis, paraffin sections were deparaffinized, rehydrated, and subjected to antigen retrieval in citrate buffer (pH 6.0) using microwave heating. Endogenous peroxidase activity was blocked with 3% hydrogen peroxide, and nonspecific binding was blocked by incubation with 5% bovine serum albumin (BSA; MCE, USA) for 30 min at room temperature. Sections were incubated overnight at 4°C with specific primary antibodies (listed in Table S1a), followed by incubation with HRP‐conjugated secondary antibodies for 1 h at room temperature. Immunoreactivity was visualized using DAB substrate, and sections were counterstained with hematoxylin.

For immunofluorescence (IF) staining, paraffin sections were deparaffinized and rehydrated. Antigen retrieval was performed by incubating the sections at 37°C for 30 min in 0.125% trypsin and 20 µg/mL proteinase K. Sections were permeabilized with 0.1% Triton X‐100 for 10 min and blocked with 5% BSA for 30 min at room temperature. Primary antibodies were applied overnight at 4°C. The sections were then incubated with Alexa Fluor 488‐ or 594‐conjugated secondary antibodies for 1 h at 37°C and counterstained with DAPI. Fluorescence images were captured using a fluorescence microscope, and quantitative analysis was performed using ImageJ software (NIH, USA).

### 4.4 Isolation, Culture, and Identification of hPDLSCs

Human periodontal ligament tissues were obtained from the middle third of extracted third molars or premolars without signs of periodontitis or caries, collected from healthy volunteers aged 18–25 at the Department of Orthodontics, Tongji Hospital, Tongji Medical College, Huazhong University of Science and Technology (Wuhan, China), under ethical approval (Approval Number: TJ‐IRB202502070). The tissues were digested in a solution containing 2 mg/mL type I collagenase (Thermo Fisher Scientific, USA) and 1 mg/mL dispase II (Yishengyuan, China) at 37°C for 1.5 h. After digestion, the suspension was centrifuged at 1000 rpm for 5 min, and the resulting cells were cultured in T‐25 flasks with α‐MEM (Boster, China) supplemented with 10% fetal bovine serum at 37°C in a humidified 5% CO_2_ incubator. The culture medium was first changed on day 5, and then every 3 days thereafter. Cells at passage 0 (P0) were subcultured, and passage 2 (P2) cells were seeded into 10 cm culture dishes for further experiments. Upon reaching 85% confluence, flow cytometry was conducted using antibodies against CD14, CD34, CD45 (negative markers), and CD73, CD90, CD105 (positive markers) to verify MSC characteristics. For trilineage differentiation, P2 hPDLSCs were induced using a commercial hMSC osteogenic, adipogenic, and chondrogenic differentiation kit (Procell, China) following the manufacturer's instructions. After 3 weeks of induction, cells were stained with Alizarin Red S, Oil Red O, and Alcian Blue to assess mineralized nodules, lipid droplets, and glycosaminoglycan formation, respectively.

### 4.5 Quantitative Real‐Time PCR Analysis

Total RNA was extracted from cells using the SteadyPure Quick RNA Extraction Kit (Accurate Biology, China) according to the manufacturer's instructions. Complementary DNA (cDNA) was synthesized from the isolated RNA using the Whole Transcriptome cDNA Synthesis Kit (Accurate Biology, AG11707). Quantitative real‐time PCR (qRT‐PCR) was performed using 2× SYBR Green Master Mix (Yeasen, China) on a LightCycler 480 Instrument II system (Roche, Switzerland) to measure gene expression levels. Primer sequences are listed in Table S1b. GAPDH was used as an internal reference gene for normalization.

### 4.6 Western Blotting

For protein analysis, hPDLSCs cultured in vitro or mouse periodontal ligament tissue samples were collected and lysed on ice using RIPA buffer supplemented with 1% protease inhibitor cocktail. The lysates were centrifuged at 12 000 × g for 20 min at 4°C, and the supernatants were collected. A quarter volume of loading buffer was added to each sample, followed by denaturation at 100°C for 5 min. Proteins were separated on 8%–12% Tris SDS‐PAGE gels and transferred onto polyvinylidene difluoride (PVDF) membranes. Membranes were blocked with 5% non‐fat dry milk for 1 h at room temperature, washed three times with TBST, and incubated overnight at 4°C with primary antibodies (listed in Table S1a). After washing, membranes were incubated with HRP‐conjugated secondary antibodies for 1 h at room temperature on a rotary shaker. Protein bands were visualized using the ImageQuant 800 imaging system (GE Healthcare, USA), and band intensities were quantified using ImageJ software (NIH, USA). The original Western blot images for all blots are provided in Figure S11.

### 4.7 Cell Viability Assay

Cell viability was assessed using the Cell Counting Kit‐8 (CCK‐8; Beyotime, China) according to the manufacturer's instructions. hPDLSCs were seeded into 96‐well plates at a density of 5 × 10^3^ cells per well in 100 µL of complete culture medium and incubated overnight at 37°C in a humidified atmosphere with 5% CO_2_. On the following day, the medium was replaced with fresh complete medium containing FFA, Fer‐1, tBHQ, or SB216763, and cells were treated for either 24 or 48 h. At the end of the treatment, 10 µL of CCK‐8 reagent was added to each well, followed by a 2‐h incubation. Absorbance at 450 nm was measured using a microplate reader (Thermo Multiskan FC, USA). Each group was tested in triplicate wells, and all experiments were independently repeated three times.

### 4.8 In Vitro Model of FFA‐Induced Lipid Peroxidation and Pharmacological Rescue in hPDLSCs

An in vitro model of lipid peroxidation was established by treating hPDLSCs with free fatty acids (FFA). The FFA stock solution was prepared by mixing oleic acid and palmitic acid at a 2:1 molar ratio and conjugating with 10% fatty acid‐free bovine serum albumin (Siduorui, China) at 70°C. This formulation was selected because oleic acid and palmitic acid are two of the most prevalent fatty acids and are widely used in combination to mimic a mixed hyperlipidemic environment in vitro. The 2:1 oleic acid/palmitic acid ratio has been commonly adopted in previous lipotoxicity models, as it induces lipid overload while reducing the excessive acute cytotoxicity caused by palmitic acid alone [70]. In addition, this formulation was chosen with reference to lipotoxicity‐based cellular models simulating metabolic syndrome‐related conditions [71, 72]. The mixture was filtered through a 0.22 µm membrane and stored at room temperature. Prior to use, the stock was pre‐warmed in a 70°C water bath and diluted with pre‐warmed complete α‐MEM medium to a final concentration of 400 µM. hPDLSCs were seeded into culture plates and incubated with the FFA working solution at 37°C in a humidified atmosphere containing 5% CO_2_ for 48 h.

To evaluate the rescue effects of different pharmacological agents, cells were first exposed to FFA for 24 h and then co‐treated for an additional 24 h with either 2 µM Ferrostatin‐1 (Fer‐1; MCE, USA), 20 µM tert‐butylhydroquinone (tBHQ; MCE, USA), or 10 µM SB216763 (MCE, USA). After treatment, cells were collected for subsequent experimental analyses.

### 4.9 Osteogenic Induction, ALP and ARS Staining

hPDLSCs were seeded in 6 well plates at a density of 2 × 10^5^ cells per well. When cell confluence reached 40% to 60%, the medium was replaced with osteogenic induction medium composed of DMEM supplemented with 10% FBS, 1% penicillin/streptomycin, 50 µg/mL ascorbic acid, and 10 mM β glycerophosphate. The medium was refreshed every 2 to 3 days.

For ALP staining, cells were cultured under osteogenic conditions for 7 days, washed with PBS, and fixed with 4% paraformaldehyde for 15 min at room temperature. Cells were then incubated with BCIP/NBT ALP staining solution (Beyotime, China) according to the manufacturer's instructions. Images were captured under identical microscope settings. Semi quantitative analysis was performed using ImageJ software by calculating the percentage of ALP positive staining area relative to the total field area from at least three randomly selected fields per well. The data presented as relative ALP activity represent normalized ALP positive areas compared with the control group.

For ARS staining, cells were cultured for 21 days under osteogenic induction, fixed with 4% paraformaldehyde for 15 min, and stained with 2% Alizarin Red S solution (Beyotime, China) at pH 4.2 for 20 min. After thorough washing, images were acquired using identical imaging parameters. For quantitative analysis, bound dye was eluted with 10% cetylpyridinium chloride (MCE, USA) for 30 min, and absorbance was measured at 562 nm using a microplate reader. The data shown as relative ARS activity represent normalized absorbance values relative to the control group.

### 4.10 ROS Detection

Intracellular ROS levels in hPDLSCs were detected using the fluorescent probe DCFH‐DA (Beyotime, China). Cells were seeded into 24‐well plates and treated as indicated, followed by incubation with 10 µM DCFH‐DA at 37°C in the dark for 20–30 min. After incubation, cells were washed three times with serum‐free medium to remove excess dye and immediately observed under an inverted fluorescence microscope (Olympus IX 71, Japan). Fluorescence intensity was quantified using ImageJ software.

For ROS detection in mouse periodontal tissues, maxillary bone samples were fixed in 4% paraformaldehyde, decalcified with 10% EDTA, embedded in paraffin, and sectioned at 5 µm thickness. Sections were stained with the ROS‐sensitive fluorescent probe dihydroethidium (DHE; Beyotime, China) according to the manufacturer's instructions. Red fluorescence signals were observed under a fluorescence microscope and quantified using ImageJ software.

### 4.11 Assessment of Lipid Peroxidation

Lipid peroxidation levels in hPDLSCs under different treatment conditions were evaluated using multiple indicators, following the manufacturers’ protocols. Lipid reactive oxygen species (ROS) accumulation was assessed with the lipid peroxidation‐specific fluorescent probe BODIPY 581/591 C11 (Beyotime, China). The content of malondialdehyde (MDA), a major end‐product of lipid peroxidation, was measured using a commercial MDA assay kit (Beyotime, China). In addition, intracellular antioxidant capacity was evaluated by quantifying glutathione (GSH) and superoxide dismutase (SOD) levels using corresponding detection kits (both from Beyotime, China). All procedures were performed according to the manufacturers’ instructions.

### 4.12 Iron Ion Detection

To assess Fe^3^^+^ deposition in mouse periodontal ligament tissues, paraffin‐embedded maxillary sections were subjected to Prussian blue (PB) staining using a commercial kit (Servicebio, China) following the manufacturer's protocol. Briefly, tissue sections were deparaffinized, rehydrated to distilled water, and then incubated with a freshly prepared working solution (equal parts of potassium ferrocyanide solution and hydrochloric acid) for 10 min. Sections were washed in distilled water (three changes), counterstained with nuclear fast red for 1 min, and dehydrated, cleared, and cover‐slipped. Blue deposits indicating Fe^3^^+^ were imaged, and the positive staining area or optical density was quantified using ImageJ software.

For quantification of iron levels in hPDLSCs, two complementary approaches were applied. Total iron content was measured using a commercial iron assay kit (Beyotime, China) following standard protocols. Labile ferrous iron (Fe^2^^+^) levels were detected using the live‐cell fluorescent probe FerroOrange (Meilunbio, China), which selectively reacts with labile Fe^2^^+^ but not Fe^3^^+^ or chelated iron. Cells were washed with serum‐free HBSS or medium, incubated with 1 µM FerroOrange working solution in HBSS (pH 7.4, 0.1% DMSO) at 37°C for 30 min in the dark. Fluorescence signals were then visualized under a fluorescence microscope, and Fe^2^^+^ fluorescence intensity was quantified using ImageJ software.

### 4.13 JC‐1 Staining

To evaluate mitochondrial membrane potential (MMP), hPDLSCs were seeded into 6‐well plates after the indicated treatments. JC‐1 Mitochondrial Membrane Potential Assay Kit (Solarbio, China) was used according to the manufacturer's instructions. Cells were incubated with freshly prepared JC‐1 staining solution at 37°C for 20 min in a cell culture incubator. After incubation, the staining solution was discarded, and cells were washed twice with JC‐1 staining buffer. Fluorescence was observed using a super‐resolution confocal microscope (LSM900 Airyscan, USA). The ratio of red (J‐aggregates) to green (J‐monomers) fluorescence intensity, indicative of mitochondrial depolarization, was quantified using ImageJ software.

### 4.14 PI/Hoechst Staining

To assess cell death and membrane integrity, hPDLSCs were stained using a PI/Hoechst double staining kit (Beyotime, China) according to the manufacturer's instructions. After treatment, cells were washed twice with phosphate‐buffered saline (PBS) and incubated with a staining solution containing PI (5 µg/mL) and Hoechst 33 342 (5 µg/mL) at 37°C for 15 min in the dark. Following incubation, cells were washed gently with PBS and immediately observed under a fluorescence microscope (Olympus IX71, Japan). Viable nuclei appeared blue, while nuclei of membrane‐compromised or dead cells showed red fluorescence. The percentage of PI‐positive cells was calculated using ImageJ software for quantitative analysis.

### 4.15 Immunofluorescence Staining

hPDLSCs after treatment were seeded onto glass coverslips (Biosharp, China). Cells were fixed with pre‐cooled 4% paraformaldehyde for 30 min, followed by permeabilization with 0.5% Triton X‐100 (Beyotime, China) for 10 min at room temperature. After washing, the cells were blocked with 2% BSA for 30 min at room temperature. Samples were then incubated with primary antibodies used in this study overnight at 4°C. The next day, cells were incubated with Alexa Fluor 488‐ or 594‐conjugated secondary antibodies at 37°C for 1 h. Nuclei were counterstained with DAPI. Images were captured using a super‐resolution confocal microscope (LSM900 Airyscan, USA). All fluorescence quantitative analyses were performed using ImageJ software. For the semi‐quantitative analysis of co‐immunofluorescence staining of CD146 and the corresponding target proteins, CD146‐positive cells were defined as the target cell population, and the mean fluorescence intensity of the target protein within CD146‐positive cells was quantified.

### 4.16 Transcriptome Sequencing

P3 hPDLSCs were divided into two groups: untreated controls and cells treated with 400 µM FFA for 48 h. Total RNA was extracted from three biological replicates per group using the MJZol RNA extraction method. RNA purity and concentration were assessed using a NanoDrop 2000 spectrophotometer, while RNA integrity was evaluated by agarose gel electrophoresis and quantified using the Agilent 5300 system to obtain RNA Integrity Numbers (RINs). Libraries were prepared with the SMART‐Seq v4 Ultra Low Input RNA Kit, followed by PCR amplification and purification. Sequencing was performed on the Illumina NovaSeq X Plus platform (Majorbio, China). The RNA‐seq data have been deposited in the NCBI Sequence Read Archive (PRJNA1336338).

### 4.17 Co‐IP and Ubiquitination Analysis

Co‐immunoprecipitation assays were performed to investigate the interaction between GSK3β and NRF2, as well as the ubiquitination status of NRF2 in hPDLSCs. Following the indicated treatments, cells were lysed on ice for 30 min using IP lysis buffer containing 50 mM Tris‐HCl, 150 mM NaCl, 1% NP‐40, 1 mM EDTA, and 1% protease inhibitor cocktail (Beyotime, China). Lysates were centrifuged at 12 000 × g for 15 min at 4°C, and the supernatants were collected. Protein concentrations were measured using a BCA assay (Beyotime, China). For each sample, 800 µg of total protein was incubated overnight at 4°C with 2 µg of anti‐NRF2 antibody or normal rabbit IgG as an isotype control. The next day, 30 µL of pre‐washed Protein A/G magnetic beads (Beyotime, China) were added and incubated for 4 h at 4°C with rotation. Beads were then washed three times with lysis buffer, and bound proteins were eluted by boiling in SDS sample buffer at 100°C for 5 min. Eluted proteins were subjected to SDS‐PAGE, transferred to PVDF membranes, and immunoblotted with reciprocal antibodies. Signals were detected using ECL, and 10% of the total lysate was used as input control. For the assessment of NRF2 ubiquitination, a similar Co‐IP procedure was performed, with subsequent immunoblotting using an anti‐ubiquitin antibody. All steps were conducted at low temperatures and under light‐protected conditions to preserve protein stability and interaction specificity. Band intensities were quantified using ImageJ software.

### 4.18 siRNA Transfection

For β‐TrCP knockdown, hPDLSCs were transfected with siBTRC (BTRC (homo)‐8945 siRNA, ID: 8945, siRNA‐1110; Crisbio, China) or negative control siRNA using Lipofectamine 3000 (Invitrogen, USA) according to the manufacturer's instructions. Briefly, cells were seeded in 6‐well plates and transfected at 50%–70% confluence with a final siRNA concentration of 50 nM. After 6 h, the medium was replaced with fresh complete medium, and cells were further incubated for 24 h before FFA treatment. Cells were then harvested for subsequent Western blot and co‐immunoprecipitation analyses. The knockdown efficiency of β‐TrCP was confirmed by Western blotting.

### 4.19 Statistical Analysis

RNA‐seq data were analyzed using R software (version 4.2.2). All other statistical analyses were performed with GraphPad Prism (version 9.0). Results are presented as mean ± standard deviation (SD). Prior to statistical comparisons, data distribution was assessed for normality using the Shapiro‐Wilk test, and homogeneity of variance was evaluated using Levene's test. Comparisons between two groups were conducted using unpaired Student's *t*‐tests when assumptions of normality and equal variance were met; otherwise, the nonparametric Mann‐Whitney U test was applied. For comparisons among three or more groups, one‐way analysis of variance (ANOVA) was employed when assumptions were satisfied, followed by Tukey's multiple comparisons test or the least significant difference (LSD) post hoc test, as appropriate. If normality or homogeneity of variance assumptions were violated, the Kruskal‐Wallis test followed by Dunn's post hoc test was used. A *p*‐value < 0.05 was considered statistically significant.

## Author Contributions

## Conflicts of Interest

The authors declare no conflict of interest.

## Supporting information




**Supporting File**: advs75157‐sup‐0001‐SuppMat.docx

## Data Availability

All data needed to evaluate the conclusions in the paper are present in the paper and/or the Supplementary Materials.
