# Supplementary material for: Hyperlipidemia Aggravates Alveolar Bone Loss via Periodontal Ligament Stem Cell Ferroptosis Through GSK3β Dependent Ubiquitin‐Mediated NRF2 Degradation
Source: Adv Sci (Weinh). 2026 Apr 7;13(36):e75157. doi: 10.1002/advs.75157 (PMC13317657; doi:10.1002/advs.75157)
Supplement: Supplementary file 1 — Supporting File: advs75157‐sup‐0001‐SuppMat.docx [file ADVS-13-e75157-s001.docx]

**Hyperlipidemia aggravates alveolar bone loss via periodontal ligament stem cell ferroptosis through GSK3β dependent ubiquitin-mediated NRF2 degradation**

Yuxiao Zhang^1,2^, Xiangyao Wang^1,2^, Yaxin Wu^1,2^, Liping Liu^1,2^, Gaoshaer Nuerlan^1,2^, Ahsawle Ozathaley^1,2^, Xiaorui Zhang^1,2^, Jinping Wang^1,2^, Bowen Yang^1,2^, Jing Mao^1,2*^, Yan Liu^3,4*^, Shiqiang Gong^1,2*^

Affiliations

^1^Department of Stomatology, Tongji Hospital, Tongji Medical College, Huazhong University of Science and Technology, Wuhan 430030, China

^2^School of Stomatology, Tongji Medical College, Huazhong University of Science and Technology & Hubei Province Key Laboratory of Oral and Maxillofacial Development and Regeneration, Wuhan 430022, China

^3^Central Laboratory, Department of Orthodontics, Peking University School and Hospital for Stomatology, 100081 Beijing, China

^4^Beijing Advanced Center of Cellular Homeostasis and Aging-Related Diseases, Institute of Advanced Clinical Medicine, Peking University, Beijing, 100091, China

*Correspondence:

Jing Mao (maojing@hust.edu.cn, orcid.org/0000-0001-6353-9992)

Yan Liu (orthoyan@bjmu.edu.cn, orcid.org/0000-0002-8193-6729)

Shiqiang Gong (gsq@hust.edu.cn, orcid.org/0000-0001-9796-4081)

**Supplementary Figures**


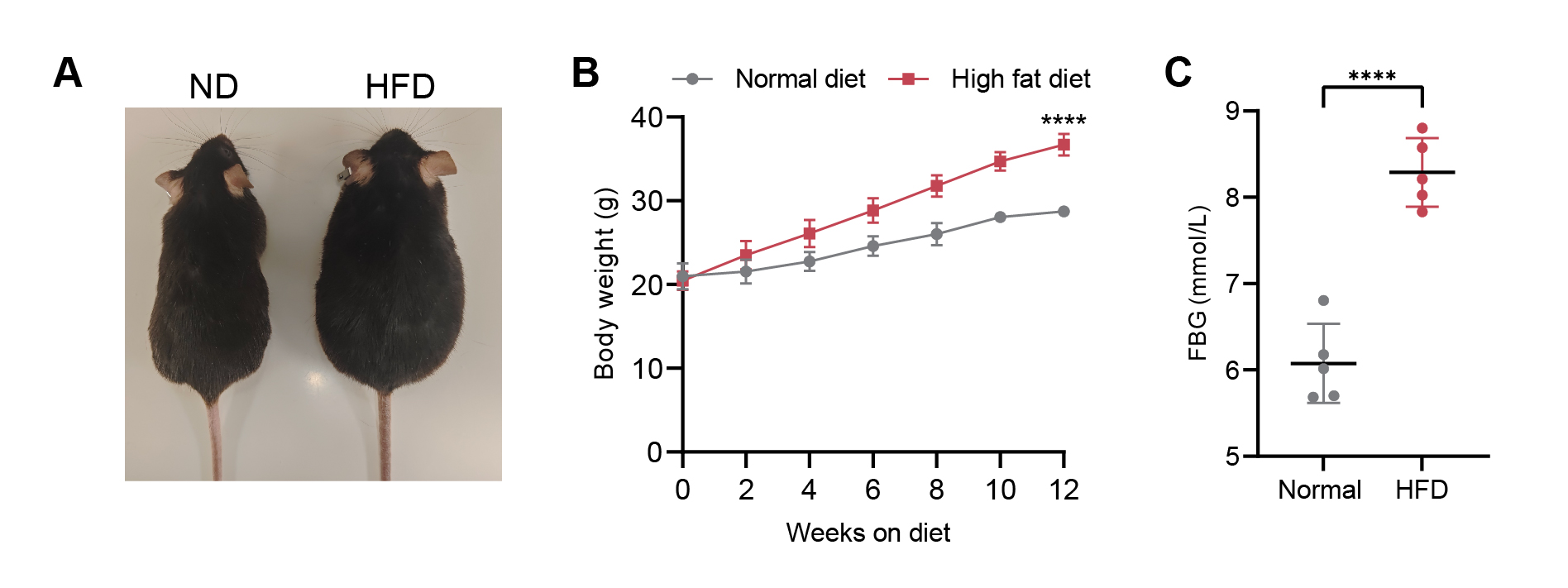


**Figure S1.** High fat diet–induced obesity phenotype and body weight gain in mice. A) Representative images showing the gross morphology of mice fed a normal diet (ND) or a high fat diet (HFD). B) Body weight changes of ND- and HFD-fed mice over the indicated feeding period. C) Fasting blood glucose (FBG) levels in ND- and HFD-fed mice after the indicated feeding period. Each dot represents an individual mouse.


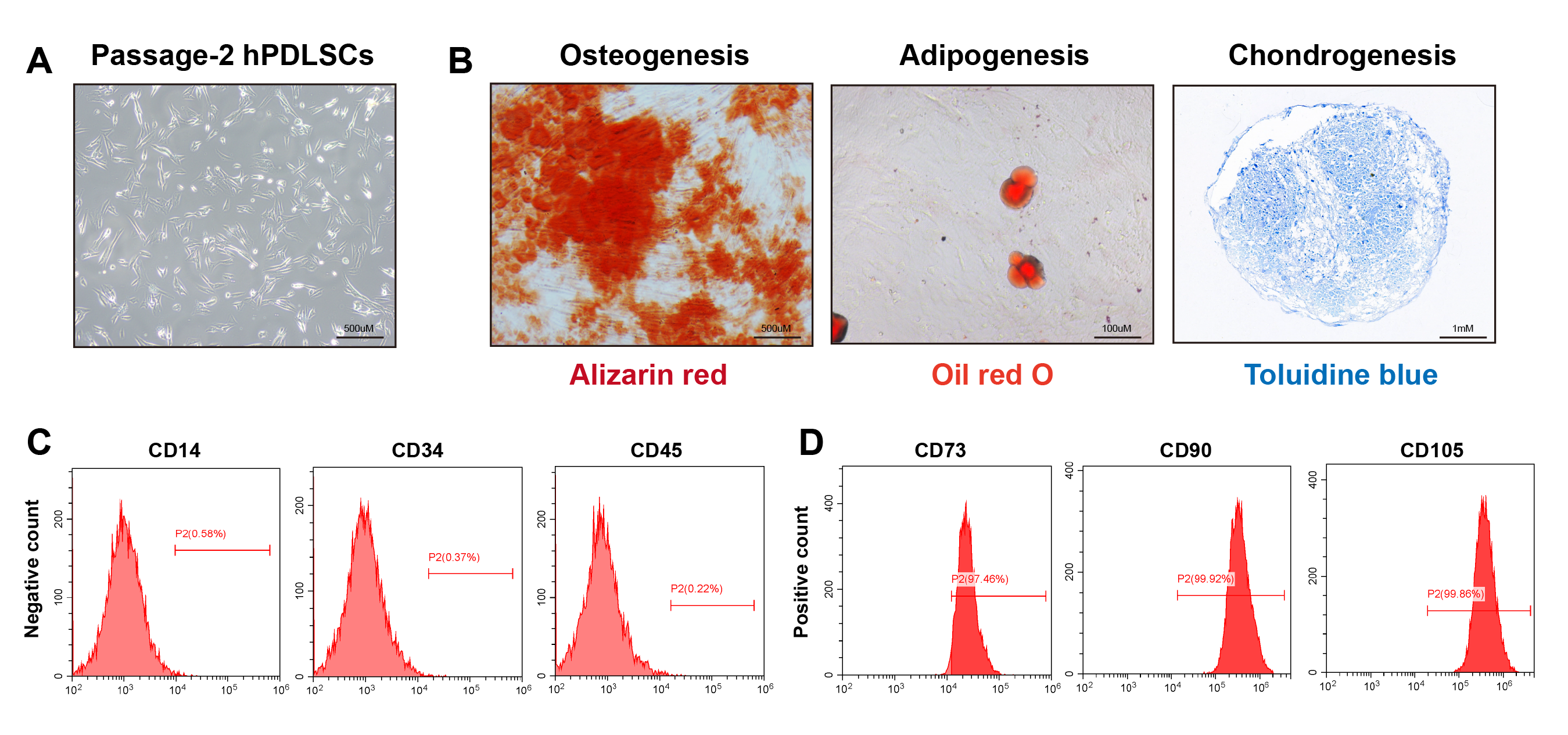


**Figure S2.** The morphology, differentiation potential, and phenotype of clinical-grade hPDLSCs were characterized. A) The morphology of hPDLSCs (P2) was shown under light microscopy. B) The multi-lineage differentiation potential of hPDLSCs was confirmed. C, D) Flow cytometry revealed that hPDLSCs were positive for mesenchymal lineage markers (CD73, CD90 and CD105), negative for hematopoietic markers (CD14, CD34 and CD45).


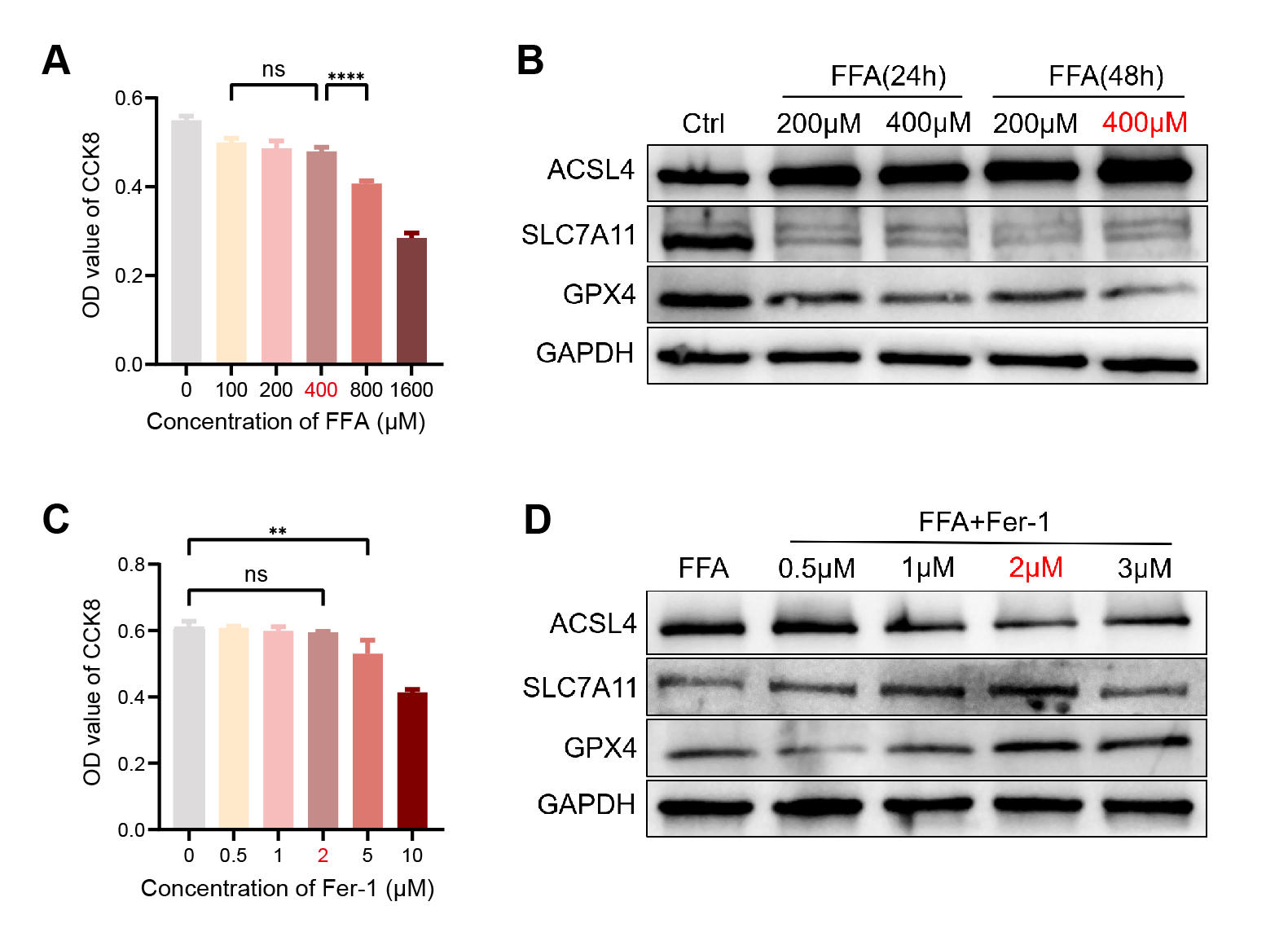


**Figure S3.** Optimization of FFA and Fer-1 concentration and treatment duration in hPDLSCs. A) Cell viability was assessed by CCK-8 assay after treatment with increasing concentrations of FFA for 48 h. B) Western blot analysis of ferroptosis-related markers in hPDLSCs treated with gradient concentrations of FFA for 24 or 48 hours. C) Cell viability was measured by CCK-8 assay after treatment with increasing concentrations of Fer-1. D) Western blot analysis of ferroptosis-related markers in hPDLSCs treated with gradient concentrations of Fer-1 for 24 hours.

**
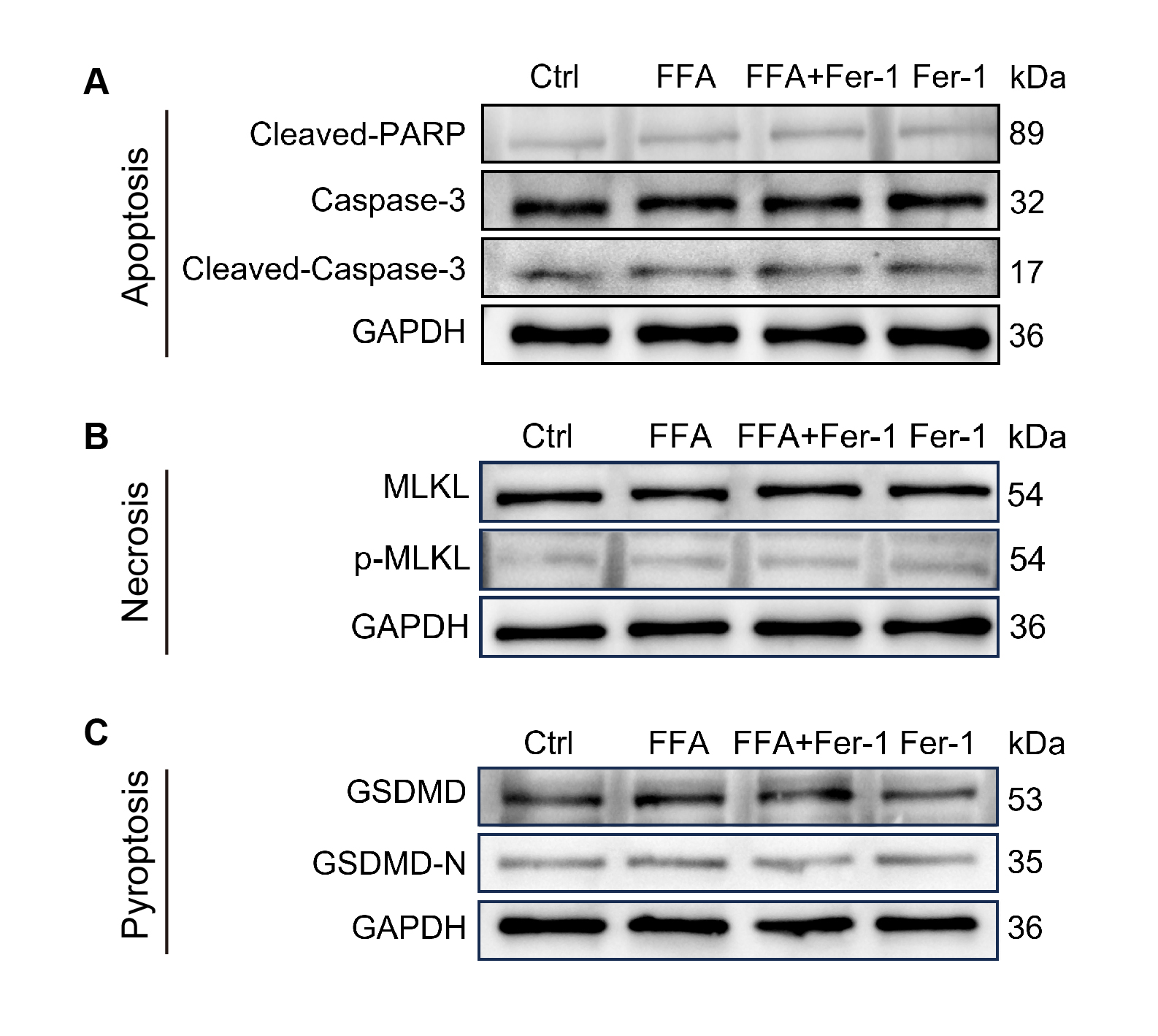
**

**Figure S4.** Assessment of apoptosis, necroptosis, and pyroptosis markers in hPDLSCs under FFA treatment. A) Western blot analysis of apoptosis-related proteins, including cleaved-PARP, Caspase-3, and cleaved-Caspase-3, in hPDLSCs treated with FFA in the presence or absence of Fer-1. B) Expression levels of necroptosis-associated proteins MLKL and phosphorylated MLKL (p-MLKL) were examined in four groups (Ctrl, FFA, FFA+Fer-1, and Fer-1). C) Pyroptosis-related proteins GSDMD and cleaved GSDMD-N were detected by Western blot analysis in four groups (Ctrl, FFA, FFA+Fer-1, and Fer-1).


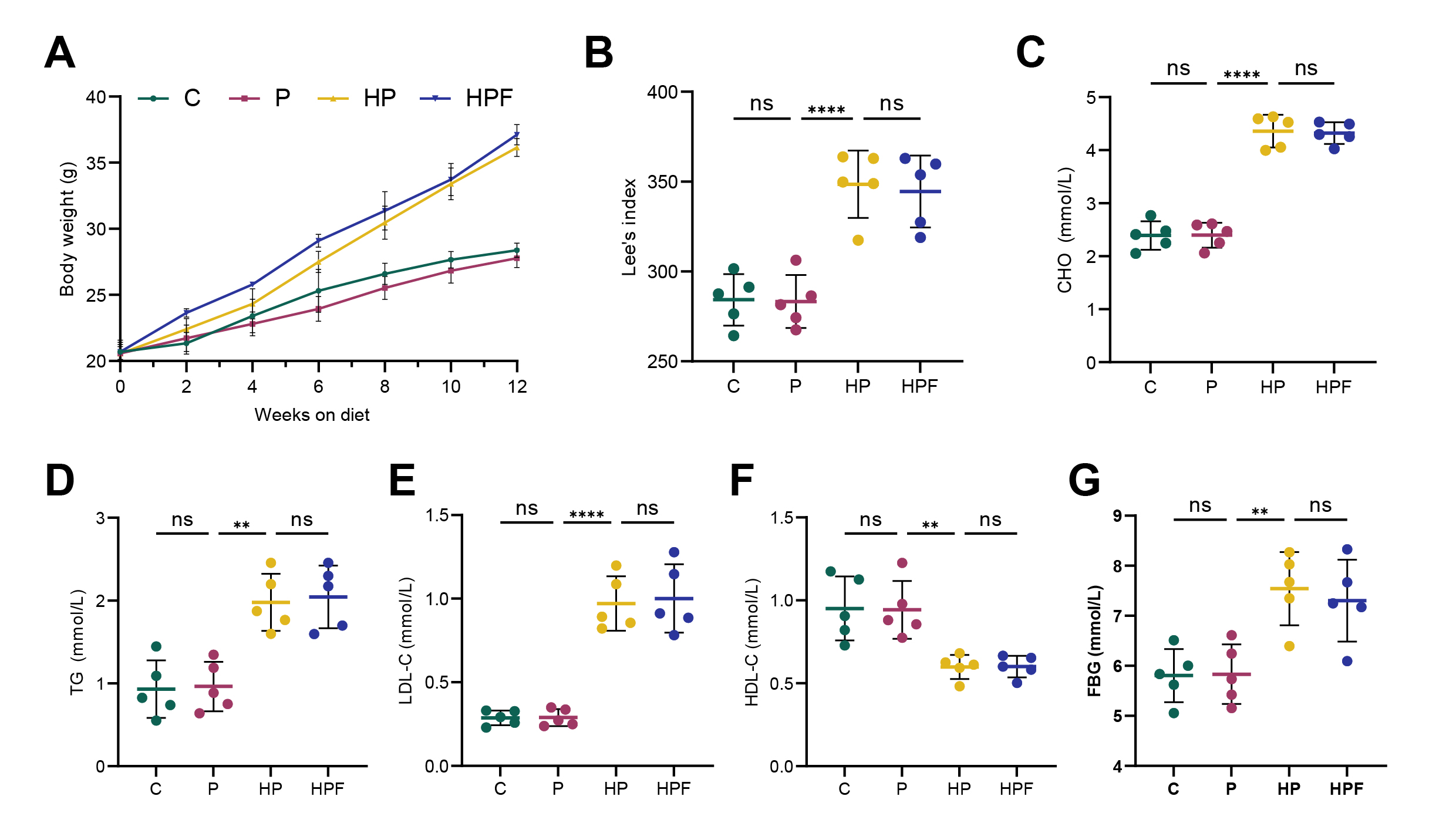


**Figure S5.** Physical measurements and blood biochemical analysis of mice in the C, P, HP, and HPF groups. A) Body weight curves of the four groups over time. B) Lee’s index at 12 weeks for each group. C-G) Serum lipid profiles, including total cholesterol (CHO), triglycerides (TG), low-density lipoprotein cholesterol (LDL-C), high-density lipoprotein cholesterol (HDL-C) and fasting blood glucose (FBG).


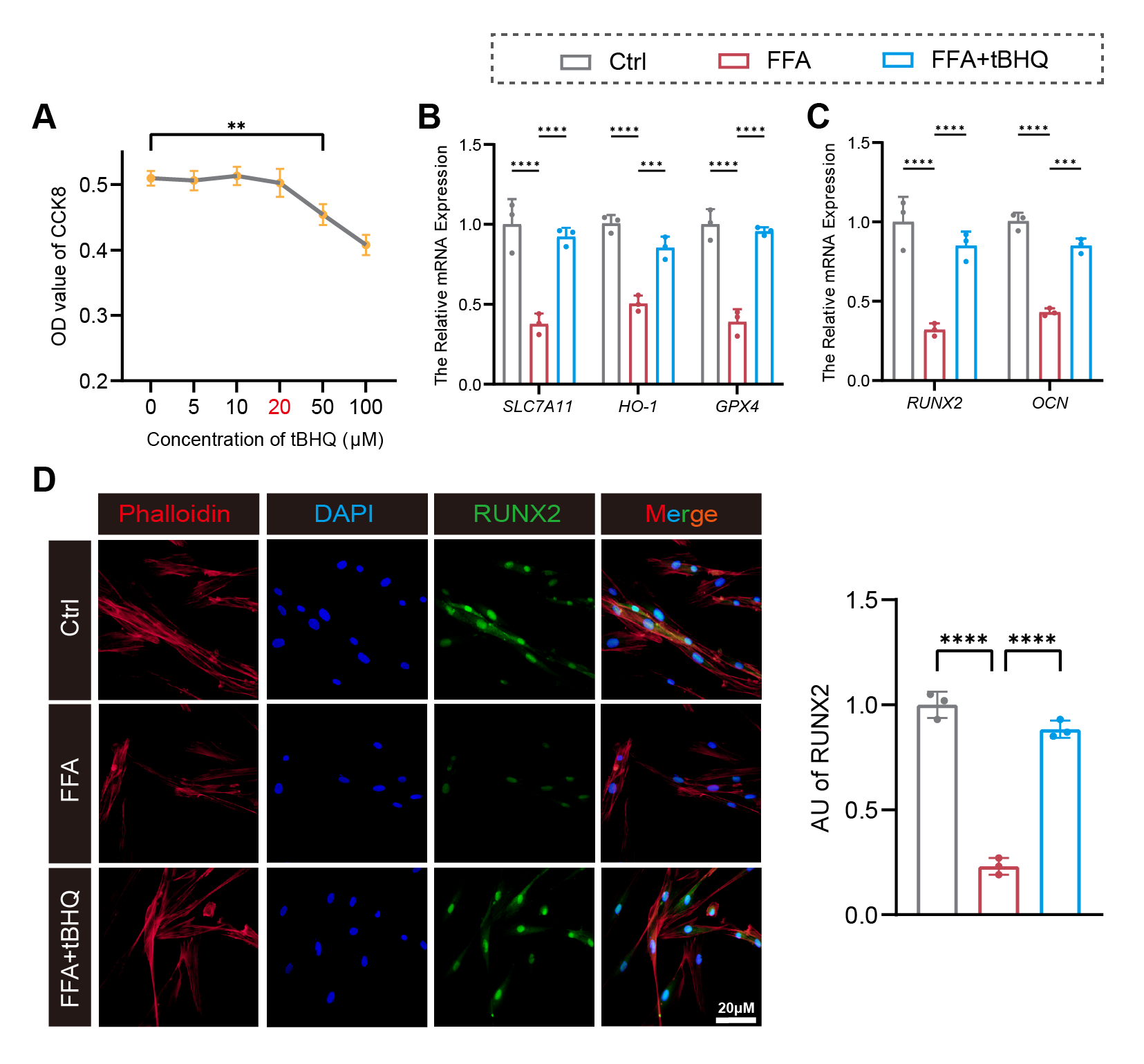


**Figure S6.** tBHQ alleviates FFA-induced ferroptosis in hPDLSCs and restores osteogenic markers. A) Cell viability was evaluated by CCK-8 assay after treatment with increasing concentrations of tBHQ. B) RT-qPCR analysis of *SLC7A11*, *HO-1*, and *GPX4* expression in the three groups. C) RT-qPCR analysis of *RUNX2* and *OCN* expression in the three groups. D) Immunofluorescence staining showing RUNX2 expression in hPDLSCs across the three experimental groups.


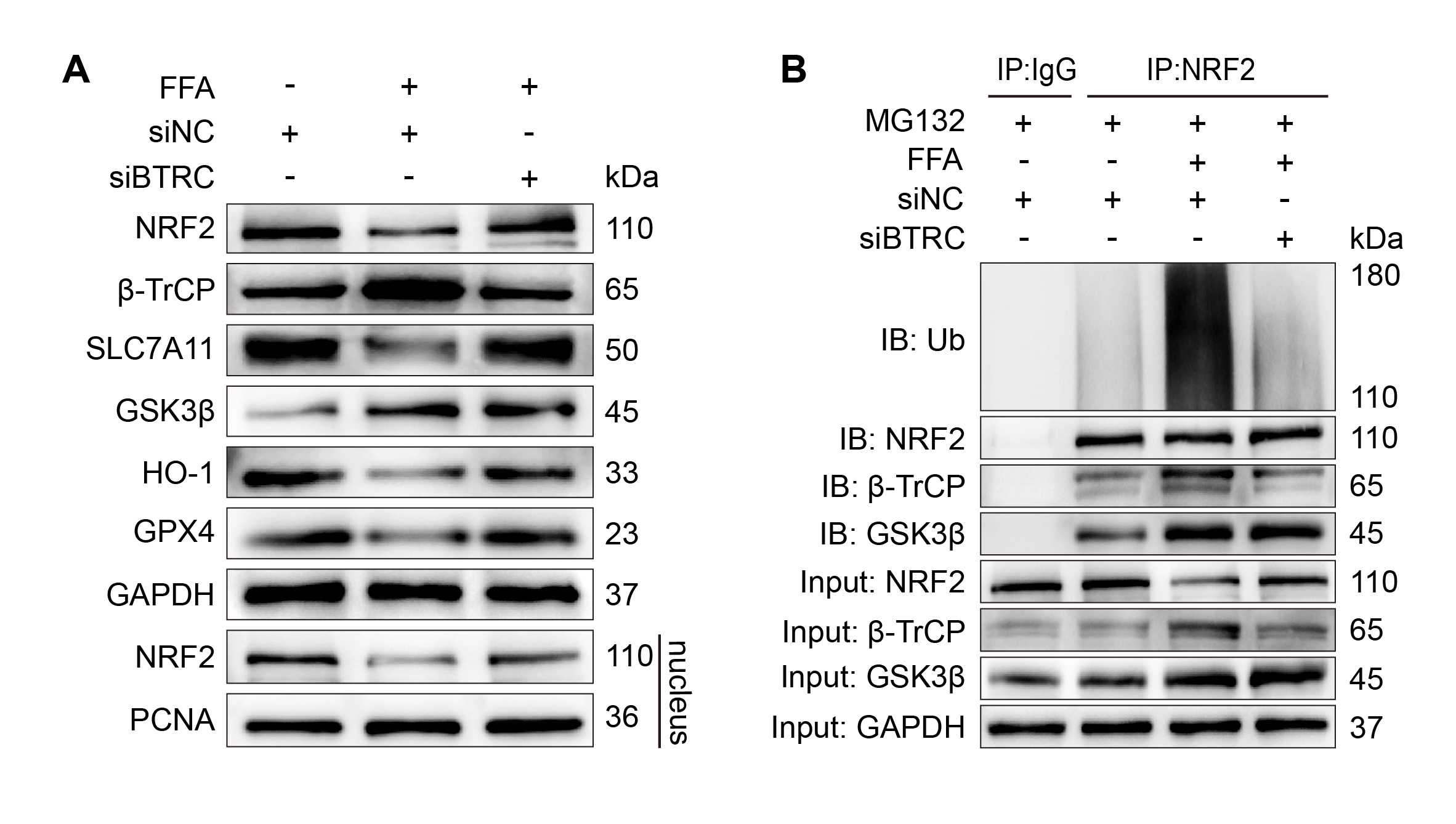


**Figure S7.** FFA-induced enhancement of GSK3β–NRF2 interaction promotes β-TrCP recognition and ubiquitin-mediated degradation of NRF2. A) Western blot analysis of NRF2, β-TrCP, SLC7A11, GSK3β, HO-1, GPX4, and nuclear NRF2 in the three indicated groups. B) Co-IP and ubiquitination assays were performed to examine the interaction of NRF2 with β-TrCP and GSK3β, as well as NRF2 ubiquitination, in the indicated groups.


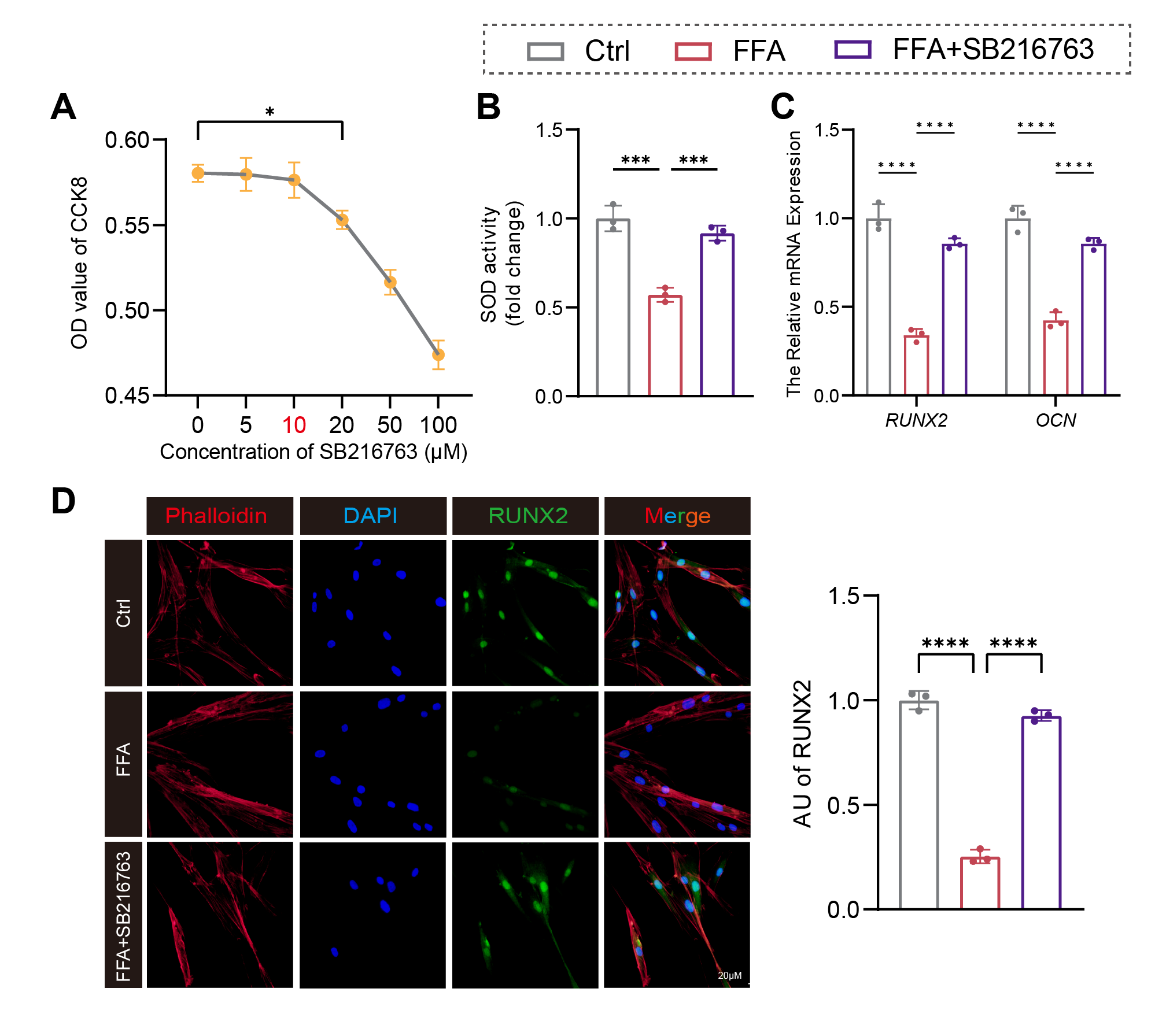


**Figure S8.** SB216763 alleviates FFA-induced ferroptosis in hPDLSCs and restores osteogenic markers. A) Cell viability was evaluated by CCK-8 assay after treatment with increasing concentrations of SB216763. B) Measurement of SOD levels in hPDLSCs to evaluate antioxidant capacity and ferroptotic stress. C) RT-qPCR analysis of *RUNX2* and *OCN* expression in the three groups. D) Immunofluorescence staining showing RUNX2 expression in hPDLSCs across the three experimental groups.

**
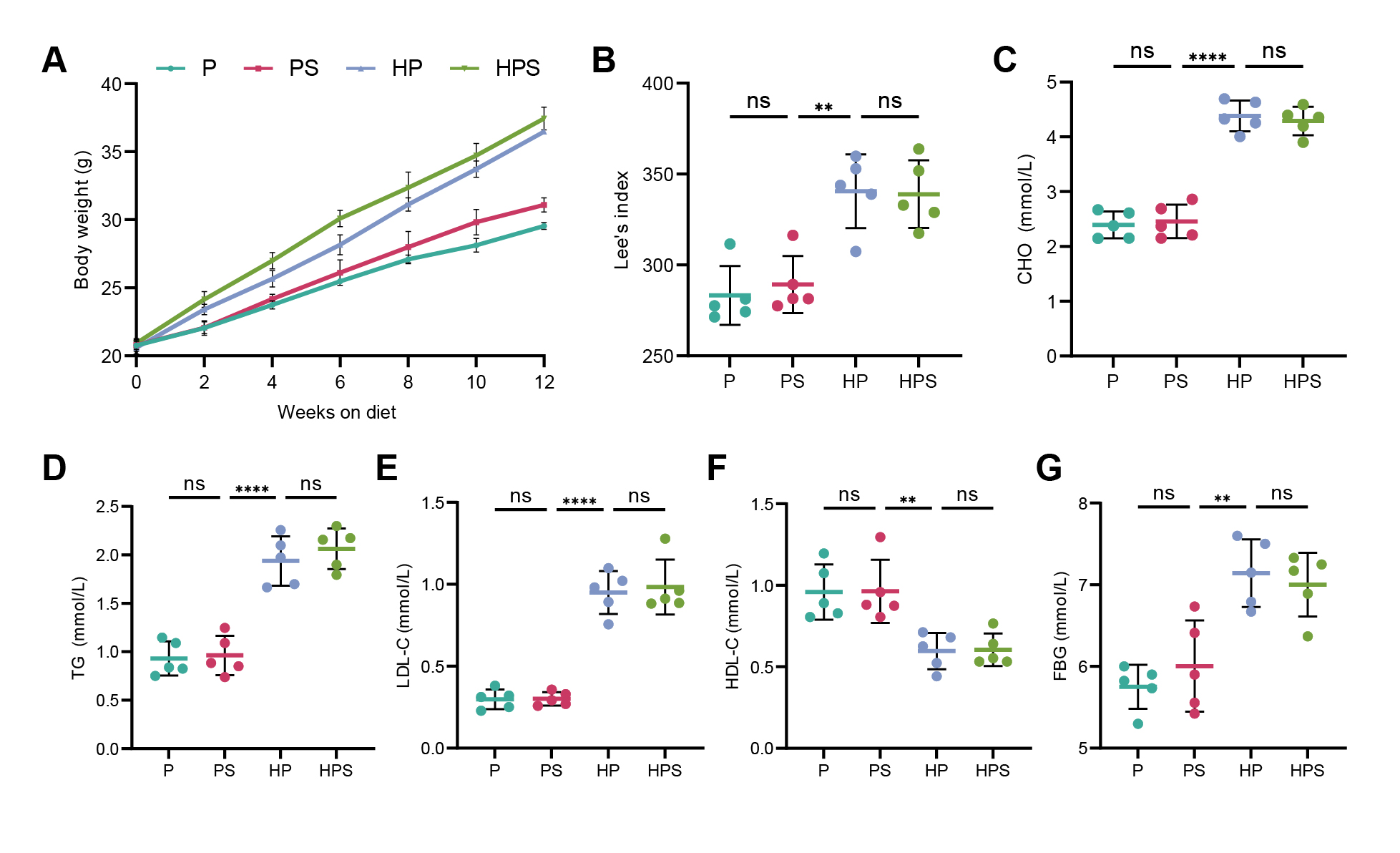
**

**Figure S9.** Physical measurements and blood biochemical analysis of mice in the P, PS, HP, and HPS groups. A) Body weight curves of the four groups over time. B) Lee’s index at 12 weeks for each group. C-G) Serum lipid profiles, including total cholesterol (CHO), triglycerides (TG), low-density lipoprotein cholesterol (LDL-C), high-density lipoprotein cholesterol (HDL-C) and fasting blood glucose (FBG).


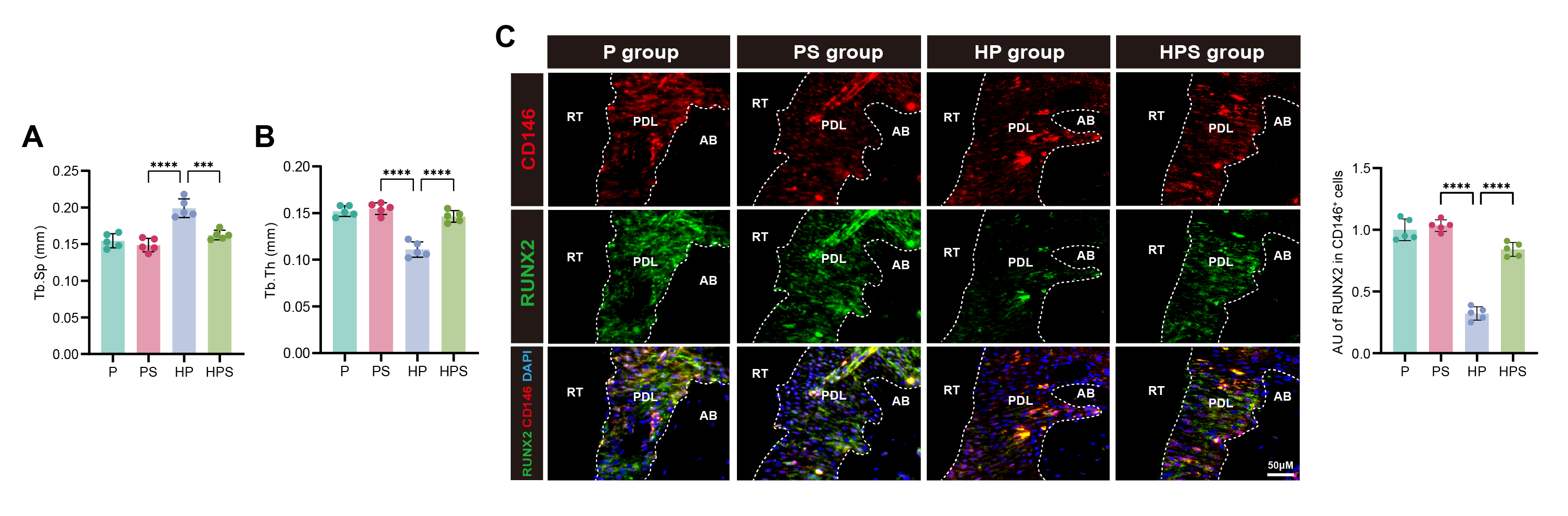


**Figure S10.** SB216763 alleviates ferroptosis of PDLSCs and alveolar bone loss in lipid metabolism–related periodontitis mice by modulating the GSK3β/NRF2/GPX4 pathway. A, B) Quantitative analysis of Tb.Sp distance and Tb.Th based on micro-CT images. C) Immunofluorescence co-staining of CD146 and GPX4 in the periodontal ligament (PDL) region, along with quantitative analysis of GPX4 fluorescence intensity in CD146⁺ cells.


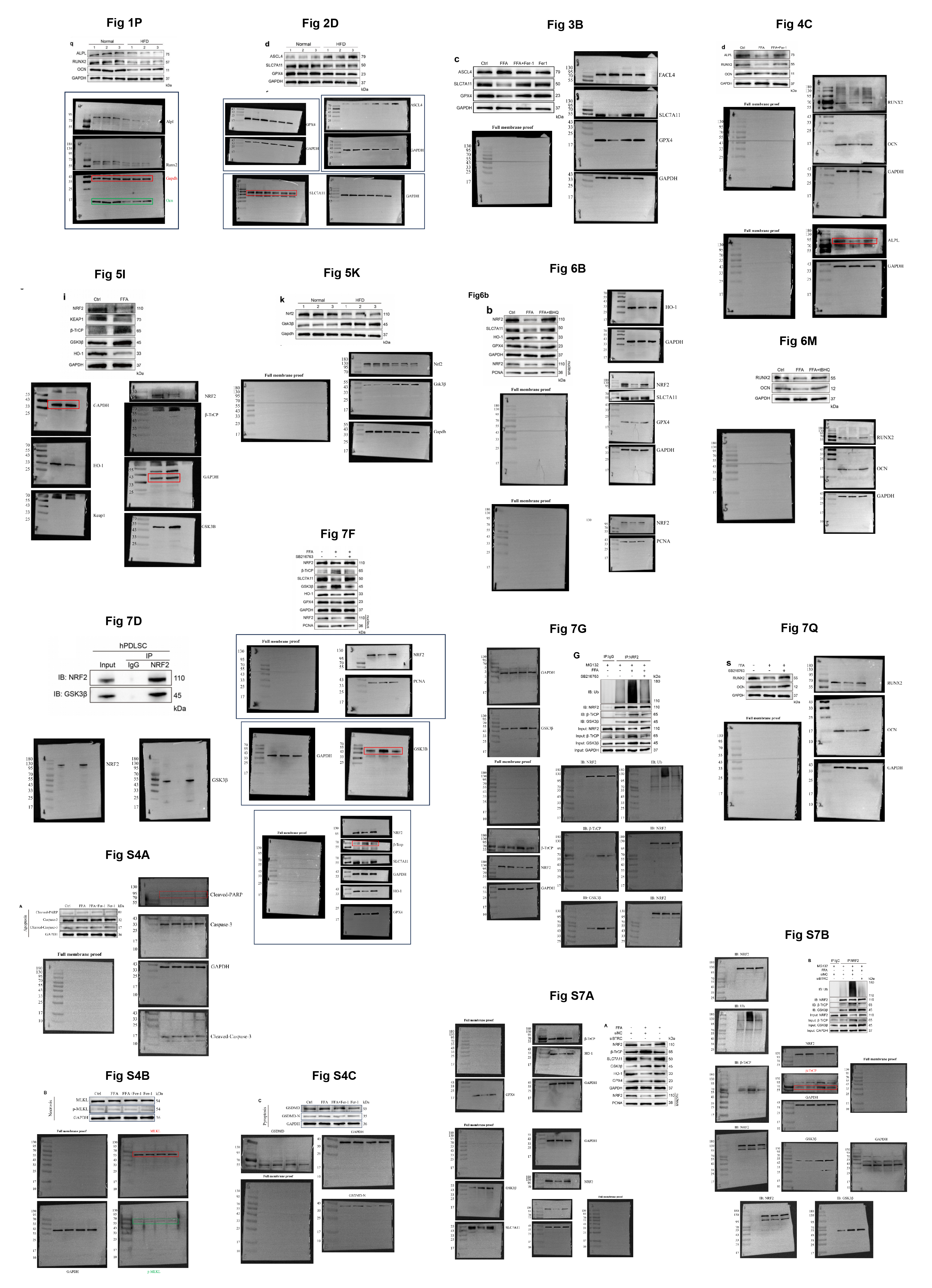


**Figure S11.** Original Western blot images.

**Supplementary Tables**

**Supplementary Table 1a. Antibodies used in this study**

| **Antibody** | **Cat No:** | **Company** | **Species** | **Application** |
| --- | --- | --- | --- | --- |
| ALPL | 11187-1-AP | Proteintech | Rabbit | WB, IHC |
| RUNX2 | 20700-1-AP | Proteintech | Rabbit | WB, IF, IHC |
| OCN | 23418-1-AP | Proteintech | Rabbit | WB, IF, IHC |
| GAPDH | 60004-1-Ig | Proteintech | Mouse | WB |
| ACSL4 | 22401-1-AP | Proteintech | Rabbit | WB |
| GPX4 | 67763-1-Ig | Proteintech | Mouse | WB, IF, IHC |
| SLC7A11 | 26864-1-AP | Proteintech | Rabbit | WB, IF, IHC |
| NRF2 | 16396-1-AP | Proteintech | Rabbit | WB, IF, IHC |
| KEAP1 | 10503-2-AP | Proteintech | Rabbit | WB |
| β-TrCP | 28393-1-AP | Proteintech | Rabbit | WB |
| GSK3β | ab32391 | Abcam | Rabbit | WB, IF, IHC |
| HO-1 | 66743-1-Ig | Proteintech | Mouse | WB, IHC |
| PCNA | 10205-2-AP | Proteintech | Rabbit | WB |
| CD146 | 17564-1-AP | Proteintech | Rabbit | IF |
| Cleaved-PARP | ET1608-10 | HUABIO | Rabbit | WB |
| Caspase-3 | ET1602-39 | HUABIO | Rabbit | WB |
| Cleaved-Caspase-3 | ET1602-47 | HUABIO | Rabbit | WB |
| MLKL | ET1601-25 | HUABIO | Rabbit | WB |
| p-MLKL | HA724063 | HUABIO | Rabbit | WB |
| GSDMD | HA601046 | HUABIO | Rabbit | WB |
| GSDMD-N | HA723254 | HUABIO | Rabbit | WB |

**Supplementary Table 1b. Primers and interference sequences used in this study**

| **Gene** | **Forward primer** | **Reverse primer** | **Purpose** |
| --- | --- | --- | --- |
| *GAPDH* | TTCAACAGCGACACCCACTC | TGGTGGTCCAGGGGTCTTAC | RT-qPCR |
| *GPX4* | AGCCCCTGGTGATAGAGAAGG | CACGCTGGATTTTCGGGTC | RT-qPCR |
| *SLC7A11* | TAGCACTGATGCCTGCACAA | GCCCATAAACACCATCTGGC | RT-qPCR |
| *ACSL4* | TCTGTTCAGCGTTTTGCAAGG | AGGGATAAACAGCAGGGCAG | RT-qPCR |
| *GSK3B* | CCGACTAACACCACTGGAAGCT | AGGATGGTAGCCAGAGGTGGAT | RT-qPCR |
| *BTRC* | GGACACAAACGAGGCATTGCCT | CAACGCACCAATTCCTCATGGC | RT-qPCR |
| *HMOX1* | CCAGGCAGAGAATGCTGAGTTC | AAGACTGGGCTCTCCTTGTTGC | RT-qPCR |
| *NFE2L2* | CACATCCAGTCAGAAACCAGTGG | GGAATGTCTGCGCCAAAAGCTG | RT-qPCR |
| *KEAP1* | CAACTTCGCTGAGCAGATTGGC | TGATGAGGGTCACCAGTTGGCA | RT-qPCR |
| *ALPL* | GCTGTAAGGACATCGCCTACCA | CCTGGCTTTCTCGTCACTCTCA | RT-qPCR |
| *RUNX2* | CCCAGTATGAGAGTAGGTGTCC | GGGTAAGACTGGTCATAGGACC | RT-qPCR |
| *BGLAP* | CGCTACCTGTATCAATGGCTGG | CTCCTGAAAGCCGATGTGGTCA | RT-qPCR |
| *CUL3* | TCGACAGCTCACACTCCAGCAT | GTGCTTCCGTGTATTAGAGCCAG | RT-qPCR |
| *NQO1* | CCTGCCATTCTGAAAGGCTGGT | GTGGTGATGGAAAGCACTGCCT | RT-qPCR |
| *BACH1* | CACCGAAGGAGACAGTGAATCC | GCTGTTCTGGAGTAAGCTTGTGC | RT-qPCR |
| *SQSTM1* | TGTGTAGCGTCTGCGAGGGAAA | AGTGTCCGTGTTTCACCTTCCG | RT-qPCR |
| *ATF4* | TTCTCCAGCGACAAGGCTAAGG | CTCCAACATCCAATCTGTCCCG | RT-qPCR |
